# Potential benefits of restrictive transfusion in upper gastrointestinal bleeding: a systematic review and meta-analysis of randomised controlled trials

**DOI:** 10.1038/s41598-023-44271-8

**Published:** 2023-10-12

**Authors:** Brigitta Teutsch, Dániel Sándor Veres, Dániel Pálinkás, Orsolya Anna Simon, Péter Hegyi, Bálint Erőss

**Affiliations:** 1 Centre for Translational Medicine, Semmelweis University, Budapest, Hungary; 2 Institute for Translational Medicine, Medical School, University of Pécs, Pécs, 7624 Hungary; 3 Department of Radiology, Medical Imaging Centre, Semmelweis University, Budapest, Hungary; 4 Department of Biophysics and Radiation Biology, Semmelweis University, Budapest, Hungary; 5 Military Hospital–State Health Centre, Budapest, Hungary; 6 First Department of Medicine, Medical School, University of Pécs, Pécs, Hungary; 7 Institute of Pancreatic Diseases, Semmelweis University, Budapest, Hungary

**Keywords:** Gastroenterology, Gastrointestinal diseases, Gastrointestinal bleeding, Upper gastrointestinal bleeding

## Abstract

The optimal red blood cell (RBC) transfusion strategy in acute gastrointestinal bleeding (GIB) is debated. We aimed to assess the efficacy and safety of restrictive compared to liberal transfusion strategies in the GIB population. We searched PubMed, CENTRAL, Embase, and Web of Science for randomised controlled trials on 15.01.2022 without restrictions. Studies comparing lower to higher RBC transfusion thresholds after GIB were eligible. We used the random effect model and calculated pooled mean differences (MD), risk ratios (RR) and proportions with 95% confidence intervals (CI) to calculate the overall effect size. The search yielded 3955 hits. All seven eligible studies reported on the upper GIB population. Restrictive transfusion did not increase the in-hospital- (RR: 0.94; CI 0.46, 1.94) and 30-day mortality (RR: 0.71; CI 0.35, 1.45). In-hospital- and 28 to 45-day rebleeding rate was also not higher with the restrictive modality (RR: 0.67; CI 0.30, 1.50; RR:0.75; CI 0.49, 1.16, respectively). Results of individual studies showed a lower rate of transfusion reactions and post-transfusion intervention if the transfusion was started at a lower threshold. A haemoglobin threshold > 80 g/L may result in a higher untoward outcome rate. In summary, restrictive transfusion does not appear to lead to a higher rate of significant clinical endpoints. The optimal restrictive transfusion threshold should be further investigated.

## Introduction

Upper gastrointestinal bleeding (UGIB) is one of the most common life-threatening emergencies, with an in-hospital mortality rate of 10%^1,2^. During the past decades, a decreasing hospitalisation trend and clinical outcome improvement have been observed due to the advances in prevention, diagnosis, and management^3,4^. However, the incidence of mortality after massive bleeding remained twofold higher compared to non-severe GIB^5^. Moreover, follow-up is crucial for this population since undesirable post-discharge outcomes remain high even after the first months^6,7^.

As part of the pre-endoscopic treatment, patients with acute blood loss often require red blood cell (RBC) transfusion^8^. Moreover, moderate and severe anaemia after UGIB are among the leading non-surgical causes of RBC transfusion^9^. Therefore, the optimal haemoglobin (Hgb) threshold for transfusion started to receive close attention^10–12^. Since starting the blood supplementation at a higher threshold (Hgb < 90–100 g/L) can lead to severe consequences due to volume overload, the benefits of restrictive transfusion (Hgb < 70–80 g/L) became an important clinical question^13^.

The American College of Gastroenterology (ACG) and European Society of Gastrointestinal Endoscopy (ESGE) Guideline strongly recommend a restrictive transfusion strategy in non-variceal UGIB (NVUGIB)^14,15^. However, these recommendations are based only on two randomised controlled trials (RCT) with different Hgb thresholds for the restrictive and liberal RBC transfusion^10,11^. The findings of Villanueva et al. played a significant role in shaping the recommendations of the American Association for the Study of Liver Diseases (AASLD) and the American Gastroenterology Association (AGA) as well. These organisations have suggested that the restrictive approach be considered for managing variceal UGIB (VUGIB)^16,17^. Meta-analyses also analysed the relationship between the transfusion modality and clinical outcomes. The article of Odatuyo et al.^18^ found a 35% and 42% decrease in mortality and rebleeding, respectively, with restrictive transfusion. However, the pooled clinical endpoints were heterogeneous among the included studies since in-hospital and more extended follow-up data were assessed together. A recent Cochrane review by Carson et al.^19^ reported results with subgroups for all types of haemorrhages. They found a decrease in 30-day mortality of patients with acute blood loss, including gastrointestinal bleeding and trauma, with restrictive transfusion.

However, it remained unanswered how the RBC transfusion affects short- and long-term outcomes after UGIB. In order to elucidate this question, we compared the different transfusion strategies, assessing the source of bleeding and transfusion thresholds separately.

## Methods

Our systematic review and meta-analysis was conducted and reported based on the recommendations of the Cochrane Handbook^20^ and the Preferred Reporting Items for Systematic Reviews and Meta-Analysis (PRISMA) 2020 guideline^21^ (Supplementary Table S1). The study protocol was registered on PROSPERO on the 14th of January, 2022, under code: CRD42022302923. Due to the limited number of studies to perform subgroup analyses with different transfusion thresholds in a pairwise setting, we deviated from the original plan to investigate by performing a proportional meta-analysis to provide data on this relevant clinical question (see “Synthesis methods”). Details of the methods can be accessed in the Supplementary Material (Supplementary Table S2).

### Inclusion criteria and search strategy

RCTs were eligible for the systematic review if they compared restrictive to liberal transfusion after an acute GIB episode and reported at least one of the following outcomes: mortality (considered as primary endpoint in our analysis), rebleeding, ischaemic- or thromboembolic events, complications, adverse events related to transfusion, need for intervention and length of hospital stay. For the definition of the two transfusion modalities, we accepted the followings: (1) RBC replacements below a pre-defined low threshold compared to a higher threshold; (2) RBC supplementation in a lower amount in comparison to a higher amount.

The comprehensive literature search was conducted in all fields of four medical databases MEDLINE (via PubMed), The Cochrane Central Register of Controlled Trials (CENTRAL), Embase, and Web of Science (15th of January, 2022). Filters and restrictions were not implemented during the search. The search strategy contained three main concepts: gastrointestinal bleeding, transfusion, and randomised controlled trials (Supplementary Table S2). To find associated articles, we manually searched the key articles, review articles, and meta-analyses' reference sections. We also read those articles which cited the eligible studies.

### Study selection and data collection

The two independent review authors (BT, DP) used reference manager software (EndNote X9, Clarivate Analytics, Philadelphia, PA, USA) for study selection. After duplicate removal, all records were screened first by title and abstract, then by full text, following a strict protocol. Cohen's kappa coefficient was calculated after each step to measure inter-rater reliability^22^. Disagreements were solved by a third investigator (BE).

Two reviewers (BT, OS) independently extracted all relevant data from the eligible articles into Excel spreadsheets (Office 365, Microsoft, Redmond, WA, USA) (Supplementary Table S2). In case of disagreement, a consensus between the two data extractors was reached by a third party's arbitration (BE). Authors of the eligible studies were contacted to provide additional outcome data.

### Synthesis methods

Due to clinical heterogeneity, the minimum number of RCTs to perform the meta-analytical calculations and subgroup analyses for the pairwise analysis (restrictive vs. liberal groups compared directly) was three. In-hospital and follow-up data (28–42 day follow-up period) was pooled separately when possible. Subgroups were made based on the source of bleeding. Due to the low number of studies, subgrouping based on transfusion threshold was not possible. For this reason, we chose the two most commonly used thresholds in the literature (70 g/L and 80 g/L) and performed a proportional meta-analysis (indirect comparison of the liberal and restrictive groups) to investigate the differences between the two groups (≤ 70 g/L, > 70 g/L; ≤ 80 g/L, > 80 g/L).

The overall results of the individual studies are summarised using forest plots. For dichotomous outcomes, pooled proportions (%) and risk ratios (RRs) from 2 × 2 tables, for continuous variables, pooled mean differences (MD) were calculated with 95% confidence intervals (CIs). To assess the overall effect estimate, we applied the DerSimonian-Laird method for the random effect model^23^. A p-value of less than 0.05 was considered statistically significant in our analysis.

The statistical heterogeneity was tested with I^2^ test, applying the Q profile method for the 95% CIs. The R programming language (R Core Team 2021, v4.1.1) was used. Further statistical details can be found in the Supplementary Material (Supplementary Table S2).

### Quality assessment and quality of the evidence

The RCTs' methodological quality was assessed separately by two reviewers (BT, OS) for each outcome, using the RoB 2 Tool by the Cochrane Collaboration^24^. When needed, a third reviewer settled the disagreements (BE). The quality of evidence for all outcomes was evaluated following the recommendations of the "Grades of Recommendation, Assessment, Development, and Evaluation" (GRADE) workgroup^25^. The Summary of Findings tables were prepared with the GRADEPro Guideline Development Tool^26^.

### Ethical approval

No ethical approval was required for this systematic review with meta-analysis, as all data were already published in peer-reviewed journals. No patients were involved in our study's design, conduct, or interpretation. The datasets used in this study can be found in the full-text articles included in the systematic review and meta-analysis.

## Results

### Study selection

**Figure 1:**
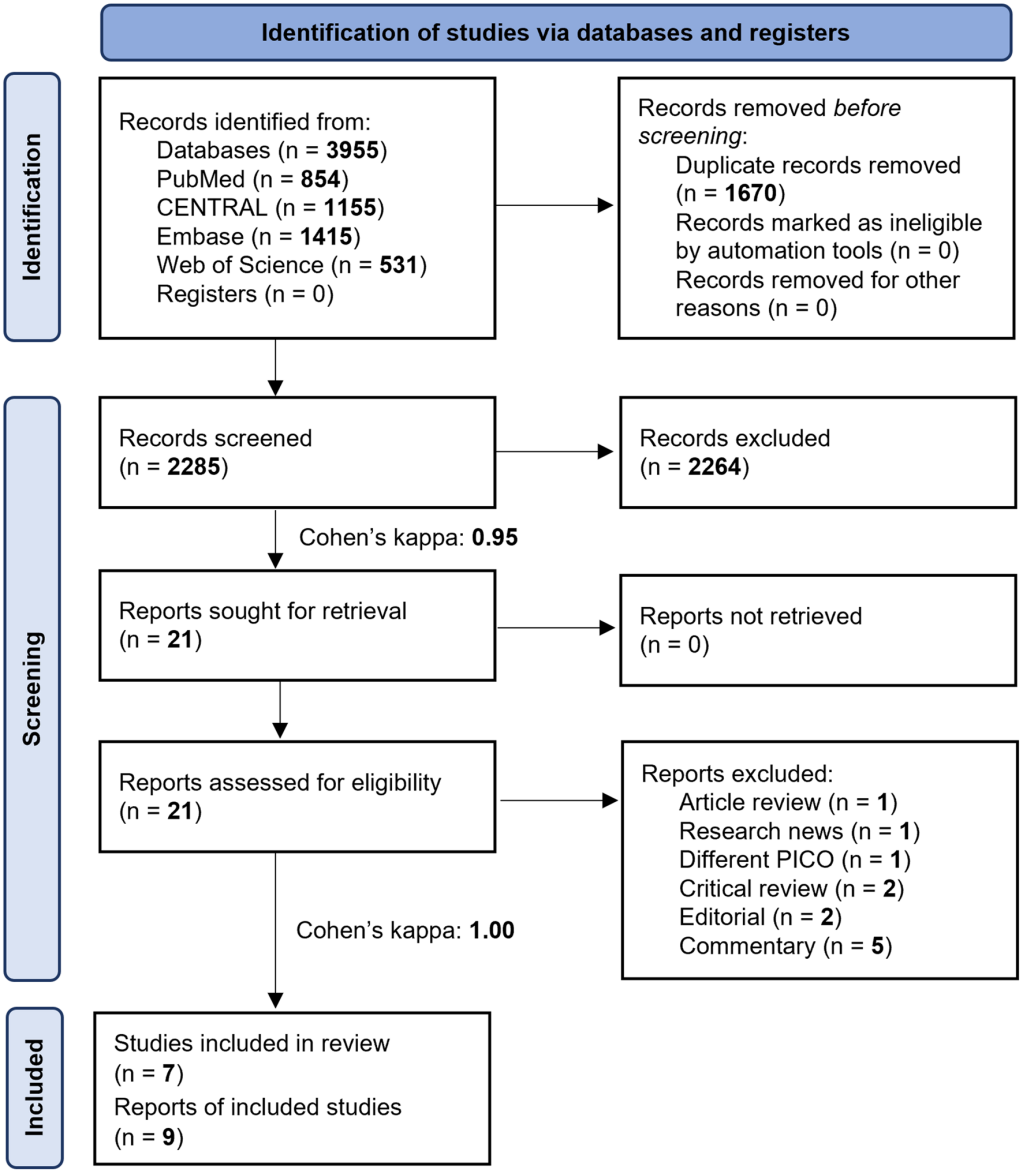
Prisma flow diagram representing the study selection process. *PICO* population-intervention-control-outcome-framework.

Altogether 3955 studies were identified by the systematic search, as outlined in the PRISMA flow diagram (Fig. 1).

Two conference abstracts, Colomo et al.^27^ and Kate et al.^28^, considered initially eligible based on full-text selection (see Reports of included studies, Fig. 1), were not included in the analysis as they were duplicates of the already included RCTs^11,12^. No ongoing trials were identified during the search. The Supplementary material lists excluded studies' characteristics (Supplementary Table S3).

### Basic characteristics of the included articles

Characteristics of the studies and study populations are detailed in Tables 1, 2. One of the seven RCTs was published as a conference abstract^29^ and one as an editorial^30^. Besides the multicentric, cluster-randomised feasibility trial of Jairath et al.^10^, all studies were parallel-group, single-centre RCTs. Exclusion criteria, reported outcomes and further details of the included studies can be found in Supplementary Table S4.

**Table 1:** Characteristics of the included randomised controlled trials.

Study characteristics													
Study	Country	Study period	Follow- up (days)	Source of bleeding	Restrictive transfusion				Liberal transfusion				Amount of RBC transfused
					Threshold Hgb (g/L) or Hct (%)	Target Hgb (g/L)	Protocol adherence (%)	Nr. of patients transfused (%)	Threshold Hgb (g/L) or Hct (%)	Target Hgb (g/L)	Protocol adherence (%)	Nr. of patients transfused (%)	
Blair et al.^31^	United Kingdom	N/D	N/D	UGIB	80	N/D	26 (100%)	5 (19%)	ND	N/D	24 (100%)	24 (100%)	Not defined
Hochain et al.^a^^30^	France	N/D	6	VUGIB	25 ± 2%^d^	N/D	N/D	43 (100%)	32 ± 2%^d^	N/D	N/D	47 (100%)	Not defined
Jairath et al.^b^^10^	United Kingdom	07.2012–03.2013	28	UGIB	80	81–100	242 (96%)	133 (33%)	100	101–120	291 (76%)	247 (46%)	At the clinician's discretion
Kola et al.^12^	India	06.2015–05.2017	45	UGIB	70	70–90	N/D	N/D	80	80–100	N/D	N/D	Not defined
Lee et al.^c^^29^	South Korea	N/D	45	NVUGIB	80	> 80	N/D	N/D	100	> 100	N/D	N/D	Not defined
Villanueva et al.^11^	Spain	06.2003–12.2009	45	UGIB	70	70–90	405 (91%)	219 (49%)	90	90–110	430 (97%)	384 (86%)	1 unit, + 1 unit if Hgb level was below the threshold
Villarejo et al.^32^	Argentina	N/D	N/D	UGIB	21%^d^	N/D	N/D	N/D	28%^d^	N/D	N/D	N/D	Not defined

*Hgb* haemoglobin, *Hct* haematocrit, *N/D* no data, *UGIB* upper gastrointestinal bleeding, *VUGIB* variceal upper gastrointestinal bleeding, *NVUGIB* non-variceal upper gastrointestinal bleeding, *SD* standard deviation.

^a^Editorial, ^b^feasibility trial, cluster randomisation, multicentric, ^c^conference abstract, ^d^haematocrit, ^e^median (interquartile range).

**Table 2:** Characteristics of the included study populations.

Population characteristics														
Study	Restrictive transfusion							Liberal transfusion						
	Number of patients	Mean age ± SD (years)	Female (%)	Mean Hgb or Hct on admission ± SD (g/L)	Variceal bleeding (%)	Peptic ulcer bleeding (%)	Mean Rockall score ± SD	Number of patients	Mean age ± SD (years)	Female (%)	Mean Hgb or Hct on admission ± SD (g/L)	Variceal bleeding (%)	Peptic ulcer bleeding (%)	Mean Rockall score ± SD
Blair et al.^31^	26	60 ± 4	9 (35%)	29 ± 1·6^d^	0	17 (65%)	N/D	24	64 ± 4	8 (33%)	28 ± 12^d^	0	19 (79%)	N/D
Hochain et al.^a^^30^	43	N/D	N/D	N/D	43 (100%)	N/D	N/D	47	N/D	N/D	N/D	47 (100%)	N/D	N/D
Jairath et al^b^^10^	403	58 ± 20	159 (39%)	119 ± 32	25 (8%)	59 (20%)	2 (1–4)^e^	553	60 ± 20	211 (40%)	115 ± 34	56 (15%)	94 (24%)	2 (1–4)^d^
Kola et al^12^	112	48 ± 15	N/D	93 ± 28	50 (45%)	17 (16%)	51 ± 1	112	50 ± 15	N/D	94 ± 23	49 (44%)	19 (17%)	52 ± 1·2
Lee et al^c^^29^	32	N/D	N/D	N/D	N/D	N/D	N/D	31	N/D	N/D	N/D	N/D	N/D	N/D
Villanueva et al.^11^	444	64 ± 16	130 (29%)	96 ± 22	101 (23%)	228 (51%)	53 ± 2	445	66 ± 15	154 (38%)	94 ± 24	109 (24%)	209 (47%)	54 ± 17
Villarejo et al.^32^	14	57 ± 13	5	N/D	N/D	N/D	N/D	13	45 ± 15	4	N/D	N/D	N/D	N/D

*Hgb* haemoglobin, *Hct* haematocrit, *N/D* no data, *SD* standard deviation.

^a^Editorial, ^b^feasibility trial, cluster randomisation, multicentric, ^c^conference abstract, ^d^haematocrit, ^e^median (interquartile range).

### Pairwise analysis—quantitative synthesis

#### Red blood cell units

**Figure 2:**
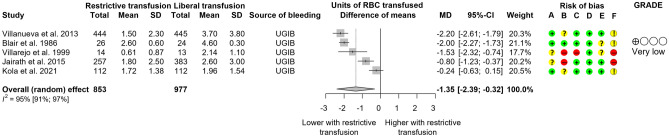
Forest plot of studies representing that restrictive transfusion resulted in fewer units of RBC transfused. *SD* standard deviation, *UGIB* upper gastrointestinal bleeding, *MD* mean difference, *CI* confidence interval. Risk of bias legend: (A) bias arising from the randomisation process, (B) bias due to deviations from intended interventions, (C) bias due to missing outcome data, (D) bias in the measurement of the outcome, (E) bias in the selection of the reported results, (F) overall bias.

Five studies^10–12,31,32^ (1830 patients) reported the amount of RBC transfusion in the UGIB population (Fig. 2). Two RCTs^11,12^ used the 70 g/L Hgb level as transfusion threshold for the restrictive group, two^10,31^ used 80 g/L, and one^32^ used the haematocrit level of 21%. A protocol for the amount of RBC transfusion needed was established in only one study^11^. Overall results showed that besides the lower transfusion threshold, patients randomised to the restrictive arm received a mean of 1.35 RBC units less (CI − 2.39, − 0.32) compared to liberal transfusion. Hochain et al.^30^ also reported that 43 participants in the restrictive group received less, a mean of 2.6 units of RBC, compared to a mean of 4.4 units in 47 participants in the liberal group.

#### In-hospital and 30-day mortality

Four RCTs published data about in-hospital mortality, including 291 participants^12,30–32^ (Fig. 3.1). Only one study^12^ enrolled more than 100 patients in both study arms. The overall result showed that restrictive transfusion did not result in an increased mortality in all included patients (RR: 0.94, CI 0.46, 1.94), nor in the UGIB subgroup (RR: 0.82, CI 0.30, 2.24). With only VUGIB, the mortality rate was higher in both arms (6/43 vs 6/47).

**Figure 3:**
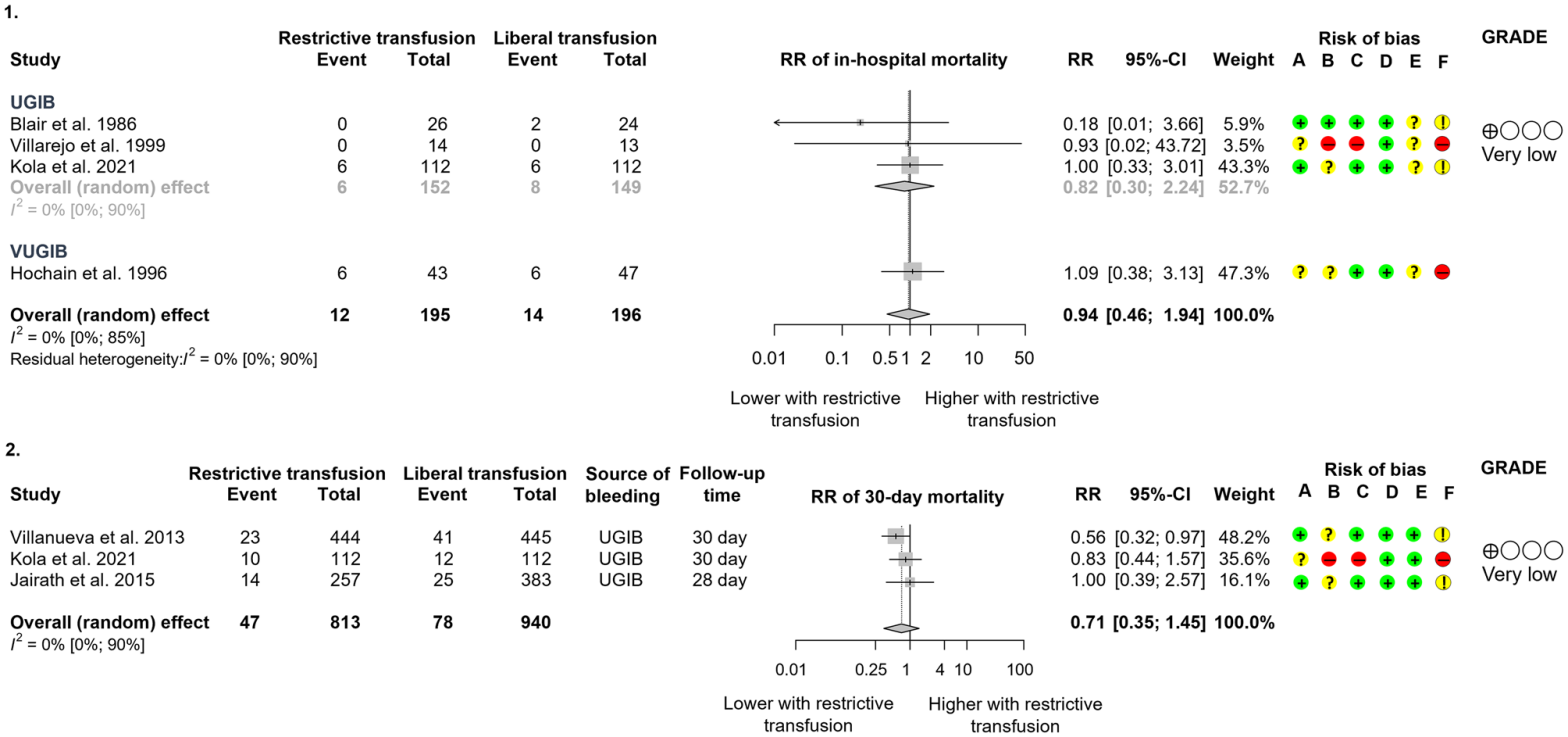
Forest plots of studies representing that (**a**) restrictive transfusion did not lead to higher in-hospital mortality compared to liberal transfusion; however, (**b**) it decreased mortality during the longer follow-up period. *RR* risk ratio, *CI* confidence interval, *UGIB* upper gastrointestinal bleeding, *VUGIB* variceal upper gastrointestinal bleeding. Risk of bias legend: (A) bias arising from the randomisation process, (B) bias due to deviations from intended interventions, (C) bias due to missing outcome data, (D) bias in the measurement of the outcome, (E) bias in the selection of the reported results, (F) overall bias.

Results from three studies^10–12^ (Fig. 3.2) with 1753 patients showed a tendency of 30-day mortality decrease, a 29% risk reduction with restrictive transfusion (RR: 0.71, CI 0.35, 1.45). There was no difference between the group regarding the 45-day survival [hazard ratio (HR): 0.83, CI 0.38, 1.85] in the RCT of Kola et al.^12^. However, in the Villanueva et al. RCT, the restrictive group had a 45% risk decrease for mortality within 45 days when the HR was adjusted for age, in-hospital bleeding, presence of cirrhosis and Rockall score (HR: 0.55, CI 0.33, 0.92).

#### In-hospital and follow-up rebleeding

Regarding in-hospital rebleeding, 1893 patients from five studies^10–12,30,31^ were analysed (Fig. 4.1). The restrictive approach did not lead to increased risk for rebleeding compared to the liberal modality (RR: 0.67, CI 0.30, 1.50). There was no statistical difference between the intervention and control groups in the UGIB subgroup (RR: 0.60, CI 0.16, 2.32). Only one study^11^, with a large sample size, proved that restrictive RBC supplementation could decrease the in-hospital rebleeding rate in a UGIB population (45/444 v. 71/445). HR adjusted for age, in-hospital bleeding, presence of cirrhosis, Rockall score, shock at admission, and baseline Hgb in the Villanueva et al. study^11^ showed a 32% and 50% risk reduction with restrictive transfusion for rebleeding from all bleeding sources (45/444 vs 71/445, HR:0.68, CI 0.47, 0.98), and from esophageal varices (10/93 v. 21/97, HR: 0.50, CI 0.23, 0.99), respectively. However, the beneficial effect of restrictive transfusion could not prevent rebleeding from peptic ulcers at a higher rate (23/228 vs 33/209, HR: 0.63, CI 0.37, 1.07) compared to liberal transfusion.

**Figure 4:**
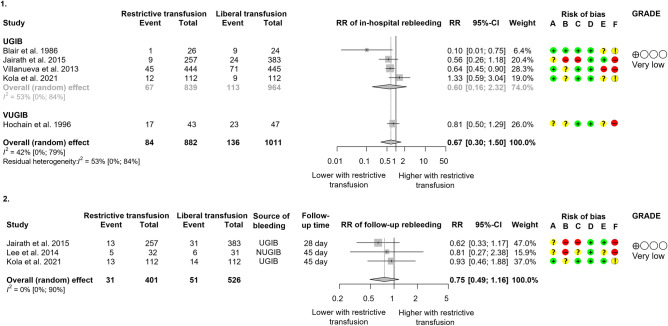
Forest plots of studies representing that restrictive transfusion did not lead to higher rebleeding during the (**a**) in-hospital and (**b**) longer follow-up period compared to liberal transfusion. *RR* risk ratio, *CI* confidence interval, *UGIB* upper gastrointestinal bleeding, *NVUGIB* non-variceal upper gastrointestinal bleeding. Risk of bias legend: (A) bias arising from the randomisation process, (B) bias due to deviations from intended interventions, (C) bias due to missing outcome data, (D) bias in the measurement of the outcome, (E) bias in the selection of the reported results, (F) overall bias.

Rebleeding 28 to 45 days post-intervention was reported in three RCTs^10,12,29^, including a total of 929 patients. The overall result showed a tendency for risk reduction on the restrictive arm; however, the result was not statistically significant (RR: 0.75, CI 0.49, 1.16) (Fig. 4.2). Regarding rebleeding from varices within 28 days, Jairath et al.^10^ reported with a high amount of missing data that 31% and 23% of participants rebled on the restrictive and liberal arm, respectively (4/13 vs 7/30).

#### Acute kidney injury

Acute kidney injury was reported in three studies^10,11,32^, including more than 1,500 patients. The overall result showed that restrictive transfusion was not inferior to liberal modality (RR: 0.79, CI 0.61, 1.03) (Supplementary Fig. S1). Moreover, there was a trend in the articles with a higher sample size of decreasing the risk of acute kidney injury in the restrictive population.

#### Length of hospital stay

Three studies^11,12,32^, including 1140 participants, provided data for length of hospital stay (Supplementary Fig. S2). The overall result did not show an increase in the length of hospital stay measured in days when patients received RBC transfusion at a lower threshold (MD: − 0.49 days, CI − 1.86, 0.89). Only in the article of Villanueva et al.^11^, was an almost 2 days shorter hospitalisation of patients enrolled on the restrictive arm (9.6 ± 8.70 vs 11.50 ± 12.80 days).

### Pairwise analysis—qualitative synthesis

#### Thromboembolic and ischemic events

Two studies^10,11^ reported the following thromboembolic events during the hospitalisation and follow-up period: myocardial infarction, acute coronary syndrome, and stroke or transient ischemic attack. In all cases, a smaller percentage of participants experienced the outcome in the restrictive arm than in the liberal group (Table 3). Villanueva et al.^11^ also reported a 39% risk reduction in acute coronary syndrome (HR: 0.61, CI 0.25, 0.49) with restrictive RBC replacement, while there was no statistical difference regarding stroke or transient ischaemic attack (HR: 0.49, CI 0.12, 2.01) between the two groups.

**Table 3:** Thromboembolic, ischemic events and post-transfusion interventions.

Outcome	Study	Number of patients		Overall risk of bias	Certainty
		Restrictive transfusion	Liberal transfusion		
Thromboembolic and ischemic events					
Thromboembolic events	Jairath et al.^10^	7/257 (2.7%)	21/383 (5.5%)	High	⨁⨁◯◯ Low
Thromboembolic events follow-up: 28 days	Jairath et al.^10^	9/257 (3.5%)	23/383 (6.0%)	High	⨁⨁◯◯ Low
Acute coronary syndrome	Villanueva et al.^11^	8/444 (1.8%)	13/445 (2.9%)	High	⨁⨁⨁◯ Moderate
Myocardial infarction	Jairath et al.^10^, Villanueva et al.^11^	2/684 (0.3%)	10/787 (1.3%)	Some concerns	⨁◯◯◯ Very low
Stroke or transient ischemic attack	Villanueva et al.^11^	3/444 (0.7%)	6/445 (1.3%)	Some concerns	⨁⨁⨁◯ Moderate
Post-transfusion interventions					
Therapeutic intervention	Jairath et al.^10^	81/257 (31.5%)	144/383 (37.6%)	High	⨁⨁◯◯ Low
Emergency surgery	Villanueva et al.^11^	4/228 (1.8%)	12/209 (5.7%)	Some concerns	⨁⨁⨁◯ Moderate
Surgical or radiological intervention	Jairath et al.^10^	10/257 (3.9%)	11/383 (2.9%)	High	⨁⨁◯◯ Low
Transjugular intrahepatic portosystemic shunt	Villanueva et al.^11^	6/139 (4.3%)	15/138 (10.9%)	Some concerns	⨁⨁⨁◯ Moderate
Balloon tamponade	Kola et al.^12^, Villanueva et al.^11^	7/251 (2.8%)	20/250 (8.0%)	Some concerns	⨁⨁⨁◯ Moderate
Second endoscopy	Villanueva et al.^11^	20/228 (8.8%)	26/209 (12.4%)	Some concerns	⨁⨁⨁◯ Moderate

#### Post-transfusion interventions

Three RCTs^10–12^ investigated the need for post-transfusion interventions. Restrictive transfusion resulted in 1% more surgical or radiological interventions in the study by Jairath et al.^10^ Regarding all other outcomes, giving RBC transfusion at a lower Hgb level resulted in a lower rate of need for intervention, especially in the case of transjugular intrahepatic portosystemic shunt and balloon tamponade application (Table 3).

#### Adverse events

In-hospital adverse events were reported in three articles^10–12^. Outcomes were less likely to occur with restrictive transfusion in each case (Supplementary Table S5).

### Proportional analysis—quantitative synthesis

Comparisons of subgroups did not reveal statistically significant differences between the lower and higher thresholds (Table 4). However, with the Hgb threshold > 80 g/L, the event rates were slightly higher compared to a threshold > 70 g/L. Forest plots can be found in the supplementary material (Figs. S3–S13).

**Table 4:** Results of proportional meta-analysis.

Outcome	Pooled event numbers (CI)	
	Threshold Hgb level ≤ 70 vs > 70	Threshold Hgb level ≤ 80 vs > 80
In-hospital mortality	0.05 (0.02, 0.14) vs 0.05 (0.02, 0.13)	Not applicable
30-day mortality	0.05 (0.03, 0.08) vs 0.07 (0.05, 0.09)	0.05 (0.04, 0.08) vs 0.07 (0.05, 0.10)
In-hospital rebleeding	0.10 (0.02, 0.34) vs 0.10 (0.04, 0.21)	0.06 (0.02, 0.18) vs 0.15 (0.06, 0.33)
28 to 42-day rebleeding	Not applicable	0.10 (0.04, 0.23) vs 0.14 (0.05, 0.32)
Acute kidney injury	0.07 (0.01, 0.34) vs 0.08 (0.01, 0.39)	0.07 (0.01, 0.33) vs 0.09 (0.01, 0.39)
	Mean (CI) days	
Length of hospital stay	6.13 (0.00, 12.30) vs 6.60 (0.42, 12.77)	5.95 (0.22, 11.69) vs 7.10 (1.27, 12.93)

*Hgb* haemoglobin, *CI* confidence intervals.

### Heterogeneity

Based on the I^2^ test, the included studies assessing mortality, follow-up rebleeding, and acute kidney injury were statistically homogenous. The statistical heterogeneity was moderate for in-hospital rebleeding and high for the number of RBC units transfused. However, these results must be interpreted cautiously due to the limited number of studies and wide CIs for the I^2^. We presume that additional subgroup analyses could not have decreased the inter-study heterogeneity, as the clinical heterogeneity of the included RCTs is high.

### Risk of bias assessment and GRADE

Differences in the baseline characteristics or missing details of the randomisation, deviations from the intended interventions, and selection of the reported results led to a moderate ("some concerns") to high risk of bias for all the included RCTs (Supplementary Figs. S15–S25).

The results of the meta-analysis had a very low level of evidence. Moderate and high risk of bias resulted in a downgrade by one- or two levels. Serious indirectness caused by the differences in the individual studies' populations, interventions, and controls also contributed to this low evidence level (Supplementary Tables S6–S10).

## Discussion

This meta-analysis was conducted to assess the efficacy and safety of restrictive transfusion over liberal transfusion after acute GIB. Our results highlighted that participants in the restrictive arms of the studies received significantly fewer units of RBCs. Though the number of patients experiencing an unfavourable outcome (in-hospital mortality, 30-day mortality, in-hospital rebleeding, 28 to 42-day rebleeding, acute kidney injury) was lower in the restrictive arm, statistically no significant differences were detected between the groups. Regarding the length of hospital stay, there were also no differences between the two transfusion modalities. Results of individual studies revealed a lower ischemic-, thromboembolic- and adverse event rate with restrictive transfusion. The comparison of transfusion threshold subgroups was not statistically significant; however, a Hgb threshold > 80 g/L may result in a higher untoward outcome rate.

The inclusion of a 30-day follow-up period in RCTs assessing the UGIB population was previously suggested by multiple expert groups^7,33^. The reasoning behind this is that different sources of bleeding tend to have different rebleeding patterns. Additionally, the exacerbation of the underlying medical condition might lead to extended hospital stays due to complications, medical procedures, and even death which might extend beyond 7 days. A previous cohort analysis also showed that GIB was associated with an increased 30-day readmission rate. Besides cardiac and respiratory decompensation and additional symptoms of anaemia, 17% of the readmissions were caused by rebleeding^34^. Proper follow-up of patients is also crucial because mortality can be more than 2.5 times higher in the 3-month post-discharge period compared to in-hospital mortality^35^. Regarding in-hospital mortality, restrictive transfusion did not perform significantly better. Still, it is essential to mention that the enrolled studies were underpowered (Table S4). In our analysis, only 200–200 patients could be investigated in both study arms with zero to only a few events, resulting in wide CIs.

On the contrary, restrictive blood transfusion showed a tendency for benefit when assessing the mortality during a more extended follow-up period with a fourfold greater sample size than for the previous outcome. The study from Villanueva et al. had the most substantial influence on the results, with a weight of around 48%, showing a 44% risk decrease with the restrictive policy^11^. However, there was only a slight difference between the restrictive and liberal groups (32/444 and 38/445, respectively) regarding 10-day mortality^11^. Based on the results from Kola et al., until the fifth day of transfusion, 4/112 and 6/112 died on the restrictive and liberal arms, respectively. In this RCT, the mortality also started to increase more than 20 days after transfusion^12^. Nevertheless, since there is a dose-dependency with the amount of RBCs transfused and adverse events^36–39^, besides the different Hgb thresholds, these dissimilarities between the interventions could also lead to inconsistent results. The highest contrast in mortality was found when participants received significantly more units of RBCs on the control arm^11^. Where patients received almost the same amount of blood, the mortality rates were similar^12^. How transfusion can influence mortality more during the follow-up than in-hospital stay is still a question. It is likely to be influenced by the underlying diseases, the amount of blood transfused, the discharge Hgb level and the cumulative complications and adverse events related to transfusion.

With the amount of transfusion, the risk of rebleeding can also increase^40^. While hypovolemia is compensated with the splanchnic vasoconstrictive response, transfusion increases the splanchnic blood pressure resulting in the erosion of the newly-formed clots and the dilution and activity reduction of clotting factors^11^. However, the existing guidelines do not support standard therapy with prothrombin complex concentrate or fresh frozen plasma^16^. Coagulation factor replacement with a low dose of vitamin K is recommended solely for haemodynamically compromised patients taking vitamin K antagonists^14^. Our results showed no statistical difference between the two transfusion modalities regarding rebleeding. However, with the restrictive policy, there was a 33% and 25% risk reduction for this outcome during the hospital stay and follow-up period, respectively. During the 28 to 45-day post-intervention period, the only significant result was from Villanueva et al.^11^, where patients on the restrictive arm had a 36% lower risk of developing in-hospital rebleeding. Here the participants received 2.2 units less transfusion on the restrictive arm. On the contrary, in the case of Kola et al.^12^, patients on the restrictive arm received only 0.2 units less blood, which could also influence the slight differences between the two groups regarding rebleeding.

Coagulopathy and thrombocytopenia can be more severe in patients with acute GIB associated with chronic liver disease and portal hypertension because of the reactive hypersplenism and impaired liver synthetic function^40^. Also, increased plasma volume can increase portal pressure, leading to recurrent variceal bleeding^11^. Analyses with variceal bleeders were reported in three RCTs^10,11,30^. Only one article, with a small sample size, randomised these patients based on this source of bleeding^30^. The other two performed subgroup analyses. Though these studies were underpowered and assessed this population only during the hospital stay, a benefit was shown with restrictive transfusion (HR: 0.50, CI 0.23, 0.99) in that article which contained the highest number of patients, 93 and 97, on the restrictive and liberal arms, respectively^11^. However, due to the high rate of complications during the first 6-weeks after the bleeding episode, this bleeding population particularly requires longer follow-up^41^.

Excessive GIB can lead to organ failure. While severe bleeding can cause pre-renal acute kidney injury, previous studies have shown that acute kidney injury is also associated with transfusion^42^. Since RBCs get damaged during storage, the cause of haemolysis with the accumulation of iron and free Hgb can lead to tissue dysfunction and kidney toxicity. Nonetheless, as evidenced by a large meta-analysis of RCTs, the prolonged storage duration of RBCs does not result in a substantial increase in the occurrence of clinically relevant outcomes, such as mortality or the need for haemodialysis^43^. Our results showed a clear tendency to reduce the risk of acute kidney injury with restrictive transfusion^42^. Only two studies reported thromboembolic and ischemic events; therefore, a meta-analysis could not be performed for these outcomes. Also, three of the involved studies even excluded the highest-risk population, with previous ischemic heart disease and cerebrovascular disease^11,12,29^.

The length of hospital stay did not show differences between the two interventions. However, this outcome is strongly influenced by the number of complications, such as rebleeding, thromboembolic or ischemic events, or adverse events linked to transfusion.

The strength of our meta-analysis is providing a comprehensive overview of the different transfusion policies with the involvement of more than 2000 patients. We could show that restrictive transfusion did not increase the occurrence of outcomes, regardless of in-hospital and more extended follow-up measurements and subgroup analyses based on the source of bleeding. However, the definition of the different transfusion thresholds differed in the included studies. Another important limitation is that only two studies^11,12^ performed sample size estimation, leading to underpowered results for both primary and secondary outcomes, which could explain our statistically non-significant findings. Also, some outcomes carried a moderate and high risk of bias. The most common reason for downgrading was that none of the studies were blinded, carrying the risk of performance and detection bias. However, addressing this issue in RCTs of this nature would present difficulties. One possible solution to reduce bias could be the implementation of a strategy where outcome assessors and data analysis are masked to the treatment allocations. Due to the indirectness and inconsistency of the studies, we had low-quality evidence for most of the results.

In practice, RBC supplementation should be carried out under optimal circumstances, paying attention to the patient's haemodynamic status and laboratory parameters to reduce the complications related to transfusion. Implementing the restrictive policy in general practice would be necessary since, besides decreasing mortality after discharge, it can also decrease hospital costs compared to liberal transfusion. In the past years, especially during the COVID-19 pandemic, there was a global shortage of RBCs^44,45^. Data from the Australian National Blood List showed that in 2022, one unit of RBCs cost around A$357^46^. Based on our results, participants on the restrictive arm had 1.35 units of RBCs less, which could decrease the costs by A$482 per participant with this transfusion modality. Also, with liberal transfusion, the complication rate increases, requiring more human and financial resources.

In future RCTs and cohorts, all patients should be followed up for longer after discharge. Since the number of variceal bleeders and peptic ulcer bleeders was different in the included studies, generalising the results is challenging. Future RCTs should also focus on these populations separately because transfusion may influence the outcomes of these patients otherwise. Lower GIB should also be investigated in this setting since no RCT was found during our systematic search. Since participants with previous ischemic and thromboembolic events were excluded in most of the included trials, the benefit of restrictive policy remains a question in these patients. Besides focusing on more homogenous populations, the Hgb thresholds for transfusion on the restrictive and liberal arms should also be investigated. The units of RBCs transfused were prospectively decided only in the study of Villanueva et al.^11^ Generally, participants on the liberal arm received more blood in each of the included studies. Previous publications have demonstrated a dose–response association between the transfused RBC units and increased non-desirable outcome events. In surgical patient groups, each additional unit of RBC led to an almost twofold increase in the odds of mortality, while the occurrence of postoperative septicemia and pneumonia from 0% with 1 unit of RBC increased to 2.29% after 3 units^47,48^. Given the limited availability of evidence for non-surgical populations, especially for GIB, the potential impact of RBC dosage on the hard clinical endpoints should be more thoroughly investigated. In future publications, it would be essential to see how the Hgb threshold only, and not the amount of transfusion, can affect the outcomes in patients suffering from GIB. Therefore, aligning with the guidance provided by transfusion guidelines, we strongly suggest the initial transfusion of a single unit of RBC in case of controlled bleeding and non-severe cases. Subsequent adjustments should be made based on the patient’s response^49^.

To summarise, restrictive transfusion, compared to the liberal modality, does not appear to lead to a higher rate of significant clinical endpoints. However, our findings must be interpreted cautiously due to the considerable uncertainty surrounding the results.

### Supplementary Information

Supplementary Information.

## Data Availability

We confirm that the data supporting the findings of this study are available within the article and its supplementary materials.
