# Supplementary material for: Potential benefits of restrictive transfusion in upper gastrointestinal bleeding: a systematic review and meta-analysis of randomised controlled trials
Source: Sci Rep. 2023 Oct 12;13:17301. doi: 10.1038/s41598-023-44271-8 (PMC10570344; doi:10.1038/s41598-023-44271-8)
Supplement: Supplementary file 1 — Supplementary Information. [file 41598_2023_44271_MOESM1_ESM.docx]

**Title**

Potential benefits of restrictive transfusion in upper gastrointestinal bleeding: a systematic review and meta-analysis of randomised controlled trials

**Authors**

Brigitta Teutsch^a,b,c^, Dániel Sándor Veres^a,d^, Dániel Pálinkás^a,e^, Orsolya Anna Simon^b,f^, Péter Hegyi^a,b,g^, Bálint Erőss^a,b,g^

**Collaborators**

Alan Colomo^h^

**Affiliations:**

1. Centre for Translational Medicine, Semmelweis University, Budapest, Hungary
2. Institute for Translational Medicine, Medical School, University of Pécs, Pécs, Hungary
3. Department of Radiology, Medical Imaging Centre, Semmelweis University, Budapest, Hungary
4. Department of Biophysics and Radiation Biology, Semmelweis University
5. Military Hospital – State Health Centre, Budapest, Hungary
6. First Department of Medicine, Medical School, University of Pécs, Pécs, Hungary
7. Institute of Pancreatic Diseases, Semmelweis University, Budapest, Hungary
8. Hospital Quirón Costa Adeje, Universitat Autònoma de Barcelona. Santa Cruz de Tenerife, Spain

**Correspondent author:**

**Dr. Bálint Erőss PhD**

Institute for Translational Medicine, Medical School, University of Pécs, Pécs, 7624, Hungary

E-mail address: [eross.balint@pte.hu](mailto:eross.balint@pte.hu)

Telephone number: +36 30 887 4028

**FIGURE AND TABLE LEGENDS**

**Figure S1.** Forest plot of studies representing that restrictive transfusion is not inferior to liberal transfusion regarding acute kidney injury. RR – risk ratio, CI – confidence interval, UGIB – upper gastrointestinal bleeding. Risk of bias legend: (A) bias arising from the randomisation process, (B) bias due to deviations from intended interventions, (C) bias due to missing outcome

**Figure S2.** Forest plot of studies representing that restrictive transfusion did not result in a more extended hospital stay (measured in days). SD – standard deviation, MD – mean difference, CI – confidence interval, UGIB – upper gastrointestinal bleeding. Risk of bias legend: (A) bias arising from the randomisation process, (B) bias due to deviations from intended interventions, (C) bias due to missing outcome data, (D) bias in the measurement of the outcome, (E) bias in the selection of the reported results, (F) overall bias.

**Figure S3.** Forest plot of studies representing pooled proportions of **in-hospital mortality** based on a haemoglobin threshold of 70 g/L. (meta-analysis)

**Figure S4.** Forest plot of studies representing pooled proportions of **in-hospital mortality** based on a haemoglobin threshold of 80 g/L. (systematic review)

**Figure S5.** Forest plot of studies representing pooled proportions of **follow-up mortality** based on a haemoglobin threshold of 70 g/L. (meta-analysis)

**Figure S6.** Forest plot of studies representing pooled proportions of **follow-up mortality** based on a haemoglobin threshold of 80 g/L. (meta-analysis)

**Figure S7.** Forest plot of studies representing pooled proportions of **in-hospital rebleeding** based on a haemoglobin threshold of 70 g/L. (meta-analysis)

**Figure S8.** Forest plot of studies representing pooled proportions of **in-hospital rebleeding** based on a haemoglobin threshold of 80 g/L. (meta-analysis)

**Figure S9.** Forest plot of studies representing pooled proportions of **follow-up rebleeding** based on a haemoglobin threshold of 70 g/L. (systematic review)

**Figure S10.** Forest plot of studies representing pooled proportions of **follow-up rebleeding** based on a haemoglobin threshold of 80 g/L. (meta-analysis)

**Figure S11.** Forest plot of studies representing pooled proportions of **acute kidney injury** based on a haemoglobin threshold of 70 g/L. (meta-analysis)

**Figure S12.** Forest plot of studies representing pooled proportions of **acute kidney injury** based on a haemoglobin threshold of 80 g/L. (meta-analysis)

**Figure S13.** Forest plot of studies representing pooled means of **length of hospital stay** based on a haemoglobin threshold of 70 g/L. (meta-analysis)

**Figure S14.** Forest plot of studies representing pooled means of **length of hospital stay** based on a haemoglobin threshold of 80 g/L. (meta-analysis)

**Figure S15.** Risk of bias assessment at study and at domain level for **units of red blood cell transfused** (meta-analysis)

**Figure S16.** Risk of bias assessment at study and at domain level for **units of red blood cell transfused** (systematic review)

**Figure S17.** Risk of bias assessment at study and at domain level for **in-hospital mortality** (meta-analysis)

**Figure S18.** Risk of bias assessment at study and at domain level for **follow-up mortality** (meta-analysis)

**Figure S19.** Risk of bias assessment at study and at domain level for **in-hospital rebleeding** (meta-analysis)

**Figure S20.** Risk of bias assessment at study and at domain level for **in-hospital rebleeding** (systematic review)

**Figure S21.** Risk of bias assessment at study and at domain level for **follow-up rebleeding** (meta-analysis)

**Figure S22.** Risk of bias assessment at study and at domain level for **follow-up rebleeding** (systematic review)

**Figure S23.** Risk of bias assessment at study and at domain level for **thromboembolic events** (systematic review)

**Figure S24.** Risk of bias assessment at study and at domain level for **post-transfusion interventions** (systematic review)

**` Figure S25.** Risk of bias assessment at study and at domain level for **adverse events** (systematic review)

**Table S1.** PRISMA checklist

**Table S2 –** Details of the Methods

**Table S3**. Characteristics of excluded studies

**Table S4.** Characteristics of included studies

**Table S5.** Adverse events

**Table S6.** Quality of evidence table for the meta-analysis

**Table S7.** Quality of evidence table for the systematic review (units of red blood cells transfused, rebleeding)

**Table S8.** Quality of evidence table for the systematic review (thromboembolic events)

**Table S9.** Quality of evidence table for the systematic review (post-transfusion interventions)

**Table S10.** Quality of evidence table for the systematic review (adverse events)

**Table S1.** PRISMA checklist

|  | **Item #** | **Checklist item** | **Location where item is reported** |
| --- | --- | --- | --- |
| **TITLE** | | |  |
| Title | 1 | Identify the report as a systematic review. | 1 |
| **ABSTRACT** | | |  |
| Abstract | 2 | See the PRISMA 2020 for Abstracts checklist. | 2 |
| **INTRODUCTION** | | |  |
| Rationale | 3 | Describe the rationale for the review in the context of existing knowledge. | 4 |
| Objectives | 4 | Provide an explicit statement of the objective(s) or question(s) the review addresses. | 4 |
| **METHODS** | | |  |
| Eligibility criteria | 5 | Specify the inclusion and exclusion criteria for the review and how studies were grouped for the syntheses. | 5 |
| Information sources | 6 | Specify all databases, registers, websites, organisations, reference lists and other sources searched or consulted to identify studies. Specify the date when each source was last searched or consulted. | 5 |
| Search strategy | 7 | Present the full search strategies for all databases, registers and websites, including any filters and limits used. | 5  Supplementary material |
| Selection process | 8 | Specify the methods used to decide whether a study met the inclusion criteria of the review, including how many reviewers screened each record and each report retrieved, whether they worked independently, and if applicable, details of automation tools used in the process. | 5 |
| Data collection process | 9 | Specify the methods used to collect data from reports, including how many reviewers collected data from each report, whether they worked independently, any processes for obtaining or confirming data from study investigators, and if applicable, details of automation tools used in the process. | 5 |
| Data items | 10a | List and define all outcomes for which data were sought. Specify whether all results that were compatible with each outcome domain in each study were sought (e.g. for all measures, time points, analyses), and if not, the methods used to decide which results to collect. | 5  Supplementary material |
|  | 10b | List and define all other variables for which data were sought (e.g. participant and intervention characteristics, funding sources). Describe any assumptions made about any missing or unclear information. | 5  Supplementary material |
| Study risk of bias assessment | 11 | Specify the methods used to assess risk of bias in the included studies, including details of the tool(s) used, how many reviewers assessed each study and whether they worked independently, and if applicable, details of automation tools used in the process. | 5 |
| Effect measures | 12 | Specify for each outcome the effect measure(s) (e.g. risk ratio, mean difference) used in the synthesis or presentation of results. | 5-6  Supplementary material |
| Synthesis methods | 13a | Describe the processes used to decide which studies were eligible for each synthesis (e.g. tabulating the study intervention characteristics and comparing against the planned groups for each synthesis (item #5)). | 5-6  Supplementary material |
|  | 13b | Describe any methods required to prepare the data for presentation or synthesis, such as handling of missing summary statistics, or data conversions. | 5-6  Supplementary material |
|  | 13c | Describe any methods used to tabulate or visually display results of individual studies and syntheses. | 5-6  Supplementary material |
|  | 13d | Describe any methods used to synthesise results and provide a rationale for the choice(s). If meta-analysis was performed, describe the model(s), method(s) to identify the presence and extent of statistical heterogeneity, and software package(s) used. | 5-6  Supplementary material |
|  | 13e | Describe any methods used to explore possible causes of heterogeneity among study results (e.g. subgroup analysis, meta-regression). | 5-6  Supplementary material |
|  | 13f | Describe any sensitivity analyses conducted to assess robustness of the synthesised results. | 5-6  Supplementary material |
| Reporting bias assessment | 14 | Describe any methods used to assess risk of bias due to missing results in a synthesis (arising from reporting biases). | Not applicable |
| Certainty assessment | 15 | Describe any methods used to assess certainty (or confidence) in the body of evidence for an outcome. | 5 |
| **RESULTS** | | |  |
| Study selection | 16a | Describe the results of the search and selection process, from the number of records identified in the search to the number of studies included in the review, ideally using a flow diagram. | 7 |
|  | 16b | Cite studies that might appear to meet the inclusion criteria, but which were excluded, and explain why they were excluded. | 7  Supplementary material |
| Study characteristics | 17 | Cite each included study and present its characteristics. | 8-9 |
| Risk of bias in studies | 18 | Present assessments of risk of bias for each included study. | 11 Figure S3-S13 |
| Results of individual studies | 19 | For all outcomes, present, for each study: (a) summary statistics for each group (where appropriate) and (b) an effect estimate and its precision (e.g. confidence/credible interval), ideally using structured tables or plots. | 10-11 |
| Results of syntheses | 20a | For each synthesis, briefly summarise the characteristics and risk of bias among contributing studies. | 10-11 |
|  | 20b | Present results of all statistical syntheses conducted. If meta-analysis was done, present for each the summary estimate and its precision (e.g. confidence/credible interval) and measures of statistical heterogeneity. If comparing groups, describe the direction of the effect. | 10-11 |
|  | 20c | Present results of all investigations of possible causes of heterogeneity among study results. | 11 |
|  | 20d | Present results of all sensitivity analyses conducted to assess the robustness of the synthesised results. | 10-11 |
| Reporting biases | 21 | Present assessments of risk of bias due to missing results (arising from reporting biases) for each synthesis assessed. | Not applicable |
| Certainty of evidence | 22 | Present assessments of certainty (or confidence) in the body of evidence for each outcome assessed. | 11 |
| **DISCUSSION** | | |  |
| Discussion | 23a | Provide a general interpretation of the results in the context of other evidence. | 12-14 |
|  | 23b | Discuss any limitations of the evidence included in the review. | 13 |
|  | 23c | Discuss any limitations of the review processes used. | 13 |
|  | 23d | Discuss implications of the results for practice, policy, and future research. | 13-14 |
| **OTHER INFORMATION** | | |  |
| Registration and protocol | 24a | Provide registration information for the review, including register name and registration number, or state that the review was not registered. | 5 |
|  | 24b | Indicate where the review protocol can be accessed, or state that a protocol was not prepared. | 5 |
|  | 24c | Describe and explain any amendments to information provided at registration or in the protocol. | 5 |
| Support | 25 | Describe sources of financial or non-financial support for the review, and the role of the funders or sponsors in the review. | 15 |
| Competing interests | 26 | Declare any competing interests of review authors. | 15 |
| Availability of data, code and other materials | 27 | Report which of the following are publicly available and where they can be found: template data collection forms; data extracted from included studies; data used for all analyses; analytic code; any other materials used in the review. | 15 |

**Methods – further details**

**Table S2 – Details of the Methods**

| **Searchkeys used in different search engines** |
| --- |
| **2022.01.15**  **PubMed:**  (gastrointestinal haemorrhage OR gastrointestinal hemorrhage OR gastrointestinal bleed* OR GI bleed* OR GIB OR UGIB OR LGIB OR ((nonvariceal OR non-variceal OR variceal OR varix OR ulcer) AND bleeding)) AND (transfusion OR red blood cell OR liberal OR restrictive) AND random* - **854**  **CENTRAL:**  (gastrointestinal haemorrhage OR gastrointestinal hemorrhage OR gastrointestinal bleed* OR GI bleed* OR GIB OR UGIB OR LGIB OR ((nonvariceal OR non-variceal OR variceal OR varix OR ulcer) AND bleeding)) AND (transfusion OR red blood cell OR liberal OR restrictive) AND random* - **1155**  **Embase:**  (‘gastrointestinal haemorrhage’ OR ‘gastrointestinal hemorrhage’ OR ‘gastrointestinal bleed*’ OR GI bleed* OR GIB OR UGIB OR LGIB OR ((nonvariceal OR ‘non-variceal’ OR variceal OR varix OR ulcer) AND bleeding)) AND (transfusion OR ‘red blood cell’ OR liberal OR restrictive) AND random* - **1415**  **Web of Science:**  (gastrointestinal haemorrhage OR gastrointestinal hemorrhage OR gastrointestinal bleed* OR GI bleed* OR GIB OR UGIB OR LGIB OR ((nonvariceal OR non-variceal OR variceal OR varix OR ulcer) AND bleeding)) AND (transfusion OR red blood cell OR liberal OR restrictive) AND random* - **531**  In total: **3955** |
| **Study selection and data collection** |
| The following data were collected: study details (first author, year of publication, study type, number of centres, leading study site, data collection, and follow-up period), exclusion criteria, characteristics of the included population (sample size, age, the proportion of female participants, source of bleeding, haemoglobin level on admission, Rockall score), threshold and target values for restrictive and liberal transfusion, amount of RBC transfused, and data regarding all relevant outcomes. |
| **Statistical analysis** |
| Since the article of Jairath et al. 2015 was a feasibility study, the authors reported results from posthoc analyses with patients with haemoglobin levels below 12 g/L on admission as well. All these analyses were adjusted for the following factors age, heart rate, systolic blood pressure, respiratory rate, haemoglobin, time since onset of symptoms, haematemesis, suspected active bleeding, syncope, suspected shock, ischaemic heart disease, respiratory disease, renal disease, liver disease, cancer, PPI, and coagulation. Since this study was an open-label, cluster-randomised trial, in order to decrease between-study heterogeneity (imbalances of participant characteristics on both study arms), we accepted these results in our meta-analysis.  Pooled RR was calculated by the Mantel-Haenszel method [Mantel and Haenszel (1959); Robins, Greenland, and Breslow (1986); Thompson, Turner, and Warn (2001)]. The exact Mantel-Haenszel method (without continuity correction) was used to handle zero events (recommended by Cooper, Hedges, and Valentine (2009); J. Sweeting, J. Sutton, and C. Lambert (2004)).  The inverse variance weighting method was used to calculate the pooled mean difference. For the outcomes where the study number was at least 5, a Hartung-Knapp adjustment (Knapp and Hartung 2003; IntHout, Ioannidis, and Borm 2014) was used. Below 5 studies, we did not apply the adjustment.  Between-study heterogeneity was described by Higgins&Thompson’s I^2^ statistics (Higgins and Thompson 2002). Where applicable, we reported the prediction intervals (i.e. the expected range of effects of future studies) of results following the recommendations of IntHout et al. (2016).  In the subgroup analysis, we used a fixed-effects “plural” model (aka. mixed-effects model). We assumed that all subgroups share a common τ2 value as we did not anticipate a high difference in the between-study heterogeneity in the subgroups, and the study number is relatively small in some subgroups.  Outlier and influence analyses were not needed to be carried out. Due to the low number of articles (less than 10 for one outcome), Egger's test and funnel plots could not be performed to assess publication bias.  We needed to analyse the proportions separately for the comparable 2 groups as the limit of groups was not the same in the studies. In this case, more results are available in the same article in separate categories. We assumed the outcomes were more similar to each other for different samples within the same study than for samples from different studies. Consequently, we used an additional random effect in our analysis using a three-level (multi-level) meta-analysis. In these analyses, we used the logit transformation of the observed proportions, and for the 0 events continuity correction, we added 0.5 to 0 and the corresponding total case value. We used inverse variance weighting with a restricted maximum likelihood method for pooling the proportions. The two groups were handled as subgroups (with the same variance). For confidence interval calculation and prediction intervals we used a t-distribution method. In some situations, we could include only few articles in one of the groups, and additionally with several 0 event counts. In these cases, no 2, nor 3 level model was useable, and we reported only the forest plots with the individual proportions.  *Meta* and *dmetar* packages were used for the analyses.  When follow-up data were not reported, we read the results from Kaplan-Meier curves using the WebPlotDigitizer v4.6. |

**RESULTS**

| **Study** | **Title** | **Reason of exclusion** |
| --- | --- | --- |
| Al-Jaghbeer et al. 2013 | Blood transfusion for upper gastrointestinal bleeding: is less more again? | Type of publication – **critical review** |
| Azoulay et. al 2013 | Focus on transfusion, bleeding and thrombosis | Type of publication – **editorial** |
| Balthazor et al. 2013 | Transfusion strategies for acute upper gastrointestinal bleeding | Type of publication – **critical review** |
| Duggan et al. 2014 | Transfusion in gastrointestinal haemorrhage: Time to change? | Type of publication – **editorial** |
| Frazer et al. 2013 | Consider this strategy for upper GI bleeds | Type of publication **commentary** |
| Madisch et al. 2013 | Restrictive transfusion strategy in upper gastrointestinal hemorrhage | Type of publication **– article review** |
| Rockall et al. 2015 | Transfusion after acute upper gastrointestinal haemorrhage | Type of publication – **commentary** |
| Rockey et al. 2014 | To transfuse or not to transfuse in upper gastrointestinal hemorrhage? That is the question | Type of publication – **commentary** |
| Rudler et al. 2014 | Transfusion strategy in gastrointestinal bleeding: Less is best? | Type of publication – **commentary** |
| Sandlow et al. 1974 | A prospective randomised study of the management of upper gastrointestinal hemorrhage | **Different PICO** – the intervention and the control group received “aggressive” vs. standard diagnostic method to identify the source of bleeding. Patients were not randomised to receive transfusion. |
| Pohl 2013 | Transfusion management in gastrointestinal hemorrhage | Type of publication – **commentary** |
| British Medical Journal 2013 | Restrictive approach to transfusion best in upper gastrointestinal bleeding | Type of publication – **research news** |

**Table S3.** Characteristics of excluded studies

**Table S4.** Characteristics of included studies

| **Study** | **Exclusion  criteria** | **Primary outcome** | **Secondary  outcomes** | **Sample size calculation** | **Blinding** |
| --- | --- | --- | --- | --- | --- |
| Blair et al. 1986 | Oesopagheal varices | Mortality, rebleeding, units of RBC transfused | | No | No |
| Hochain et al. 1996 | Not available | Units of RBC transfused, mortality in hospital, rebleeding in hospital, complications | | No | No |
| Jairath et al. 2015 | Exsanguinating haemorrhage | Feasibility measures, 28-day mortality, 28-day rebleeding, in-hospital rebleeding, myocardial infarction, acute kidney injury, units of RBC transfused | | No | No |
| Kola et al. 2021 | “Massive exsanguinating bleeding, lower GI bleed, acute coronary syndrome, symptomatic peripheral vasculopathy, stroke, transient ischemic attack, transfusion within the past 90 days or a recent history of trauma or surgery” | 45-day mortality | In-hospital mortality,45-day rebleeding, in-hospital rebleeding, length of hospital stay, balloon tamponade, major transfusional reaction, units of RBC transfused | Yes – for mortality | No |
| Lee et al. 2014 | Liver cirrhosis, ischemic heart disease, cerebrovascular disease | Rebleeding | Clinical symptoms | Not reported | Not reported |
| Villanueva et al. 2013 | “Massive exsanguinating bleeding, acute coronary syndrome, symptomatic peripheral vasculopathy, stroke, transient ischemic attack, or transfusion within the previous 90 days; a recent history of trauma or surgery; lower gastrointestinal bleeding; a previous decision on the part of the attending physician that the patient should avoid specific medical therapy; and a clinical Rockall score of 0 with a hemoglobin level higher than 12 g per deciliter” | 45-day mortality | In-hospital rebleeding, balloon tamponade, TIPS, second endoscopy therapy, emergency surgery, adverse events, stroke or TIA, myocardial infarction, acute coronary syndrome, acute kidney injury, bacterial infections, length of hospital stay, units of RBC transfused | Yes – for mortality | No |
| Villarejo et al. 1999 | Not defined | Length of hospital stay | In-hospital mortality, renal failure, units of RBC transfused | No | No |


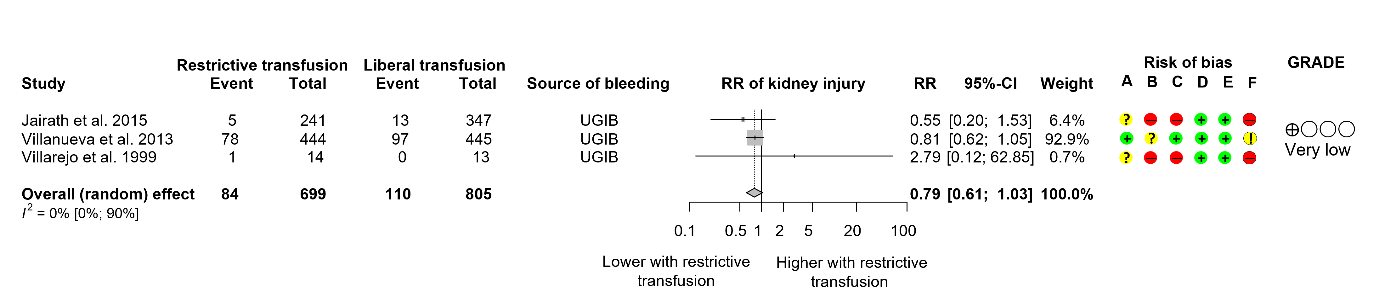


**Figure S1.** Forest plot of studies representing that restrictive transfusion is not inferior to liberal transfusion regarding acute kidney injury. RR – risk ratio, CI – confidence interval, UGIB – upper gastrointestinal bleeding. Risk of bias legend: (A) bias arising from the randomisation process, (B) bias due to deviations from intended interventions, (C) bias due to missing outcome

**
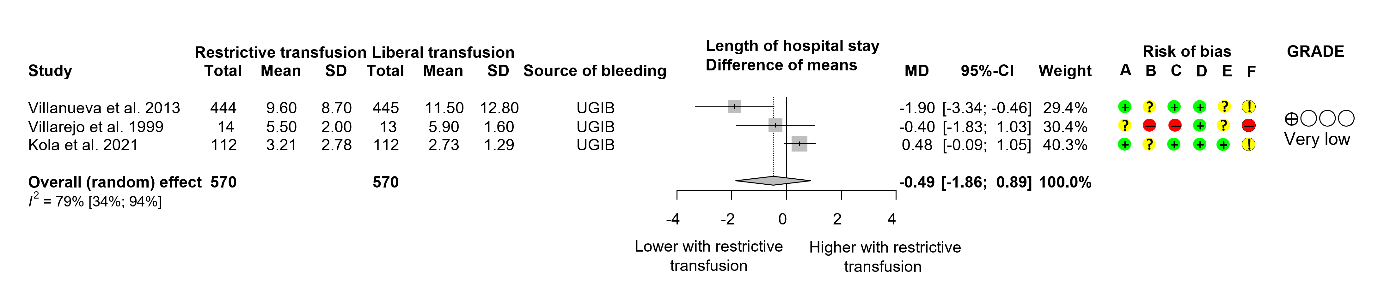
**

**Figure S2.** Forest plot of studies representing that restrictive transfusion did not result in a more extended hospital stay (measured in days). SD – standard deviation, MD – mean difference, CI – confidence interval, UGIB – upper gastrointestinal bleeding. Risk of bias legend: (A) bias arising from the randomisation process, (B) bias due to deviations from intended interventions, (C) bias due to missing outcome data, (D) bias in the measurement of the outcome, (E) bias in the selection of the reported results, (F) overall bias.


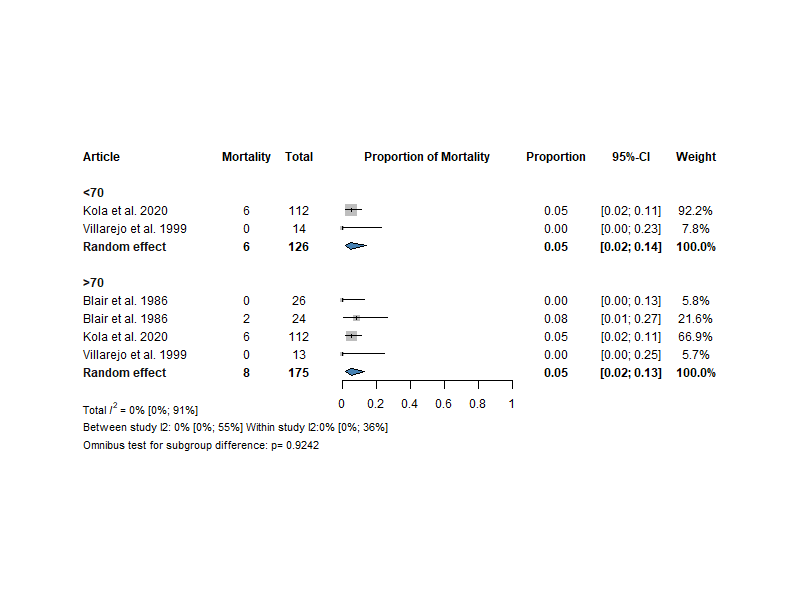


**Figure S3.** Forest plot of studies representing pooled proportions of in-hospital mortality based on a haemoglobin threshold of 70 g/L.

**Figure S4.** Forest plot of studies representing pooled proportions of in-hospital mortality based on a haemoglobin threshold of 80 g/L.

**Figure S5.** Forest plot of studies representing pooled proportions of follow-up mortality based on a haemoglobin threshold of 70 g/L.

**
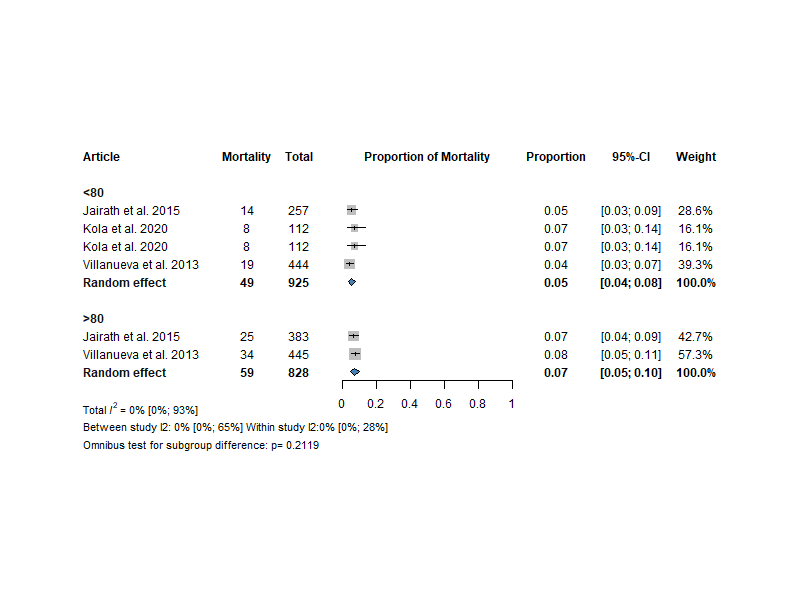
**

**Figure S6.** Forest plot of studies representing pooled proportions of follow-up mortality based on a haemoglobin threshold of 80 g/L.

**Figure S7.** Forest plot of studies representing pooled proportions of in-hospital rebleeding based on a haemoglobin threshold of 70 g/L.

**
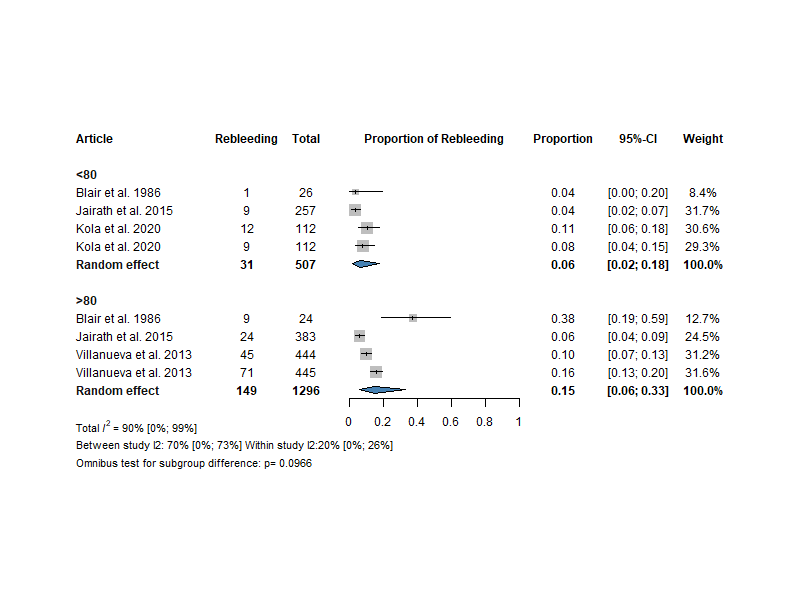
**

**Figure S8.** Forest plot of studies representing pooled proportions of in-hospital rebleeding based on a haemoglobin threshold of 80 g/L.


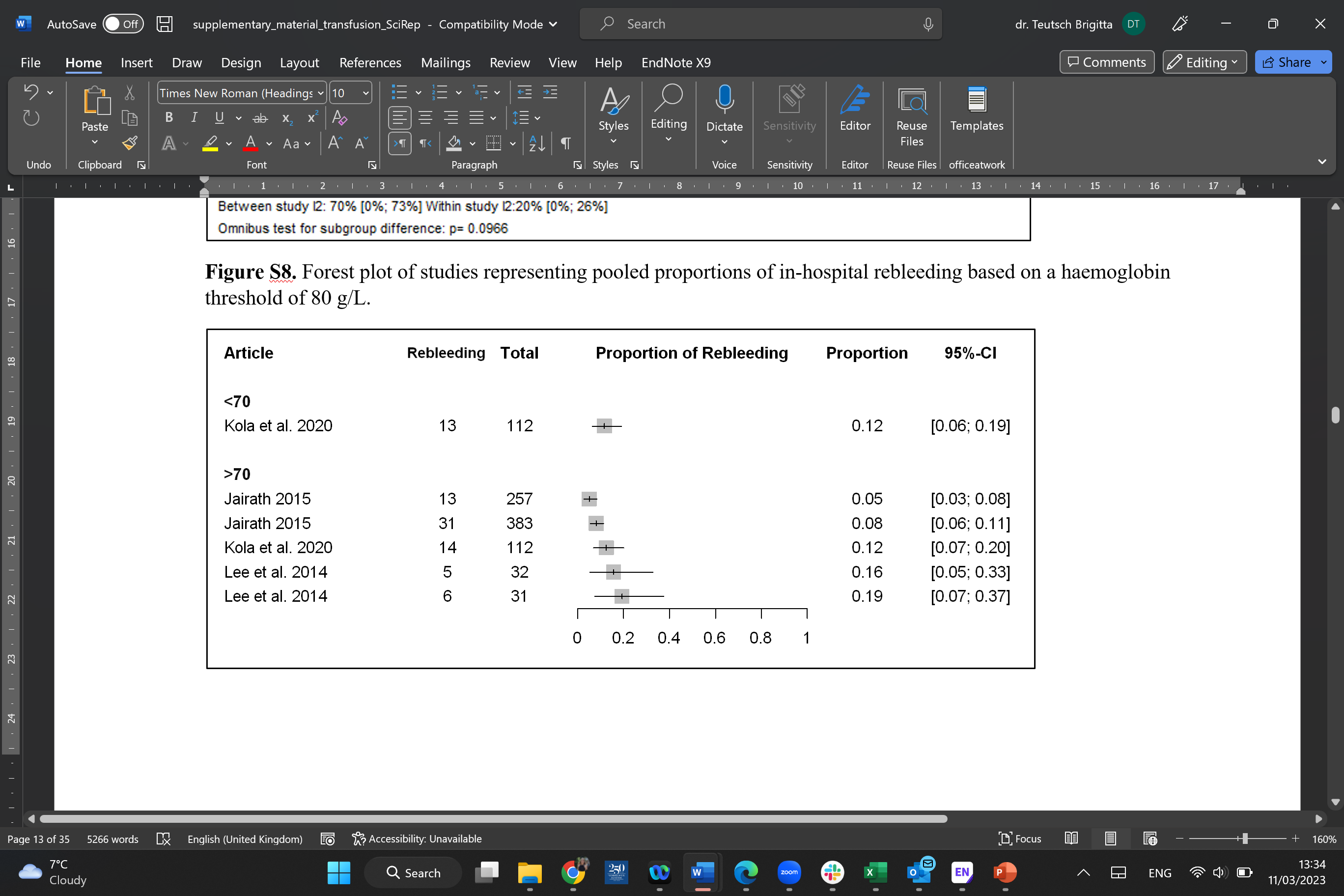


**Figure S9.** Forest plot of studies representing pooled proportions of follow-up rebleeding based on a haemoglobin threshold of 70 g/L.

**
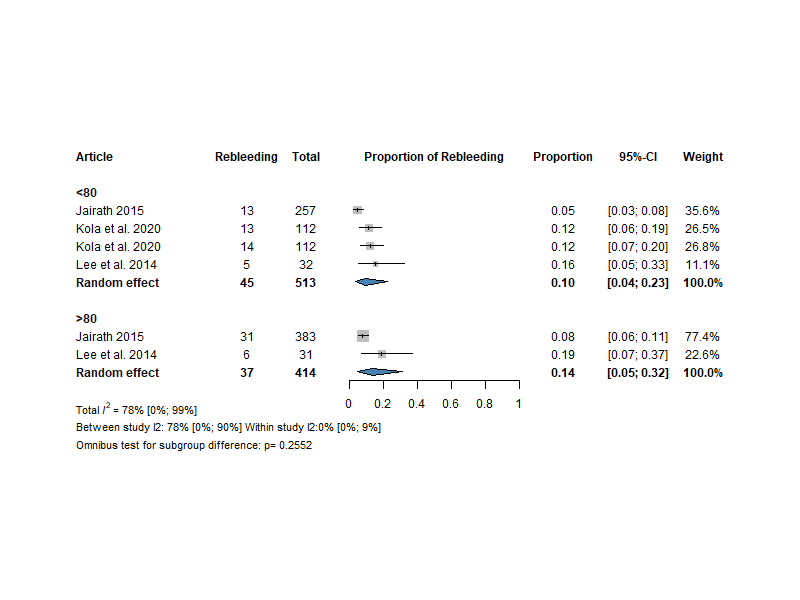
**

**Figure S10.** Forest plot of studies representing pooled proportions of follow-up rebleeding based on a haemoglobin threshold of 80 g/L.

**Figure S11.** Forest plot of studies representing pooled proportions of acute kidney injury based on a haemoglobin threshold of 70 g/L.

**Figure S12.** Forest plot of studies representing pooled proportions of acute kidney injury based on a haemoglobin threshold of 80 g/L.

**Figure S13.** Forest plot of studies representing pooled means of length of hospital stay based on a haemoglobin threshold of 70 g/L.

**Figure S14.** Forest plot of studies representing pooled means of length of hospital stay based on a haemoglobin threshold of 80 g/L.

**Table S5.** Adverse events

| Outcome | Study | Number of patients | | Overall risk of bias | Certainity |
| --- | --- | --- | --- | --- | --- |
|  |  | **Restrictive transfusion** | **Liberal transfusion** |  |  |
| Acute transfusion reaction | Kola et al. 2021^10^,  Jairath et al. 2015^8^ | 2/369 (0.5%) | 9/495 (1.8%) | High | ⨁⨁◯◯ Low |
| Serious adverse event | Jairath et al. 2015^8^ | 45/257 (17.5%) | 83/383 (21.7%) | High | ⨁⨁◯◯ Low |
| Adverse event | Villanueva et al. 2013^9^ | 179/444 (40.3%) | 214/445 (48.1%) | Some concerns | ⨁⨁⨁◯ Moderate |
| Infection | Jairath et al. 2015^8^, Villanueva et al. 2013^9^ | 186/701 (26.5%) | 227/828 (27.4%) | High | ⨁⨁⨁◯ Moderate |

**Risk of bias assessment**


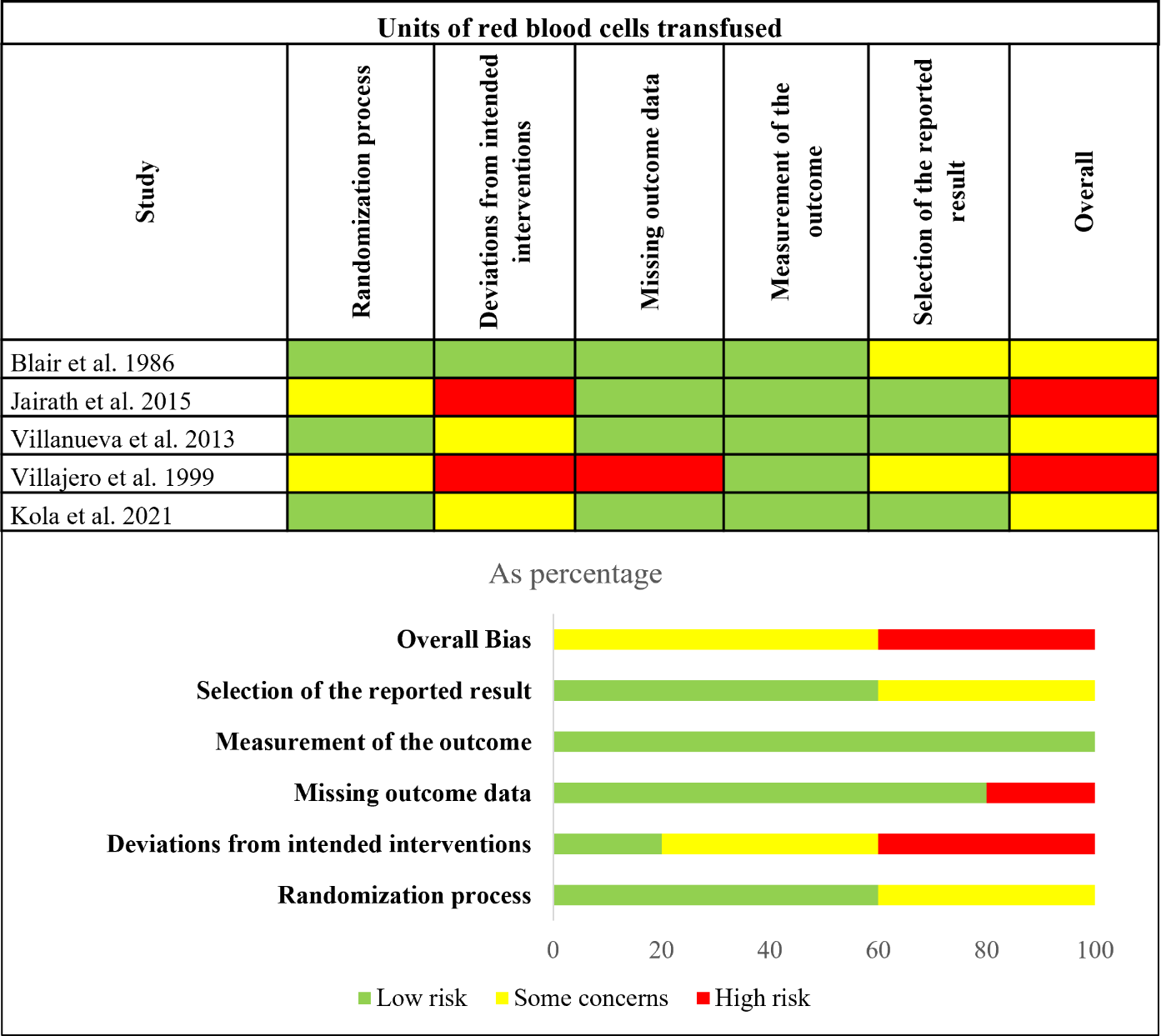


**Figure S15.** Risk of bias assessment at study and at domain level for **units of red blood cell transfused** (meta-analysis)


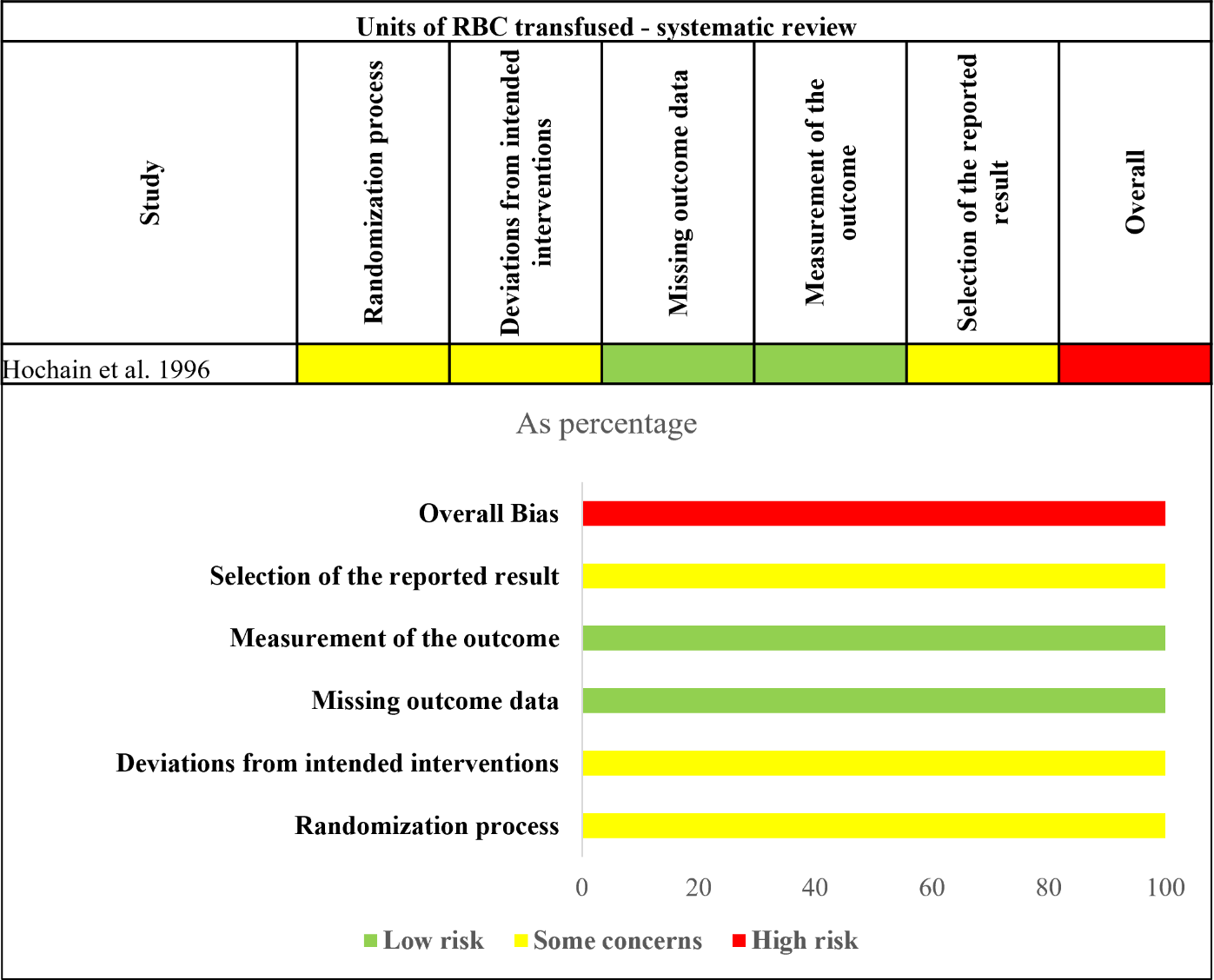


**Figure S16.** Risk of bias assessment at study and at domain level for **units of red blood cell transfused** (systematic review)


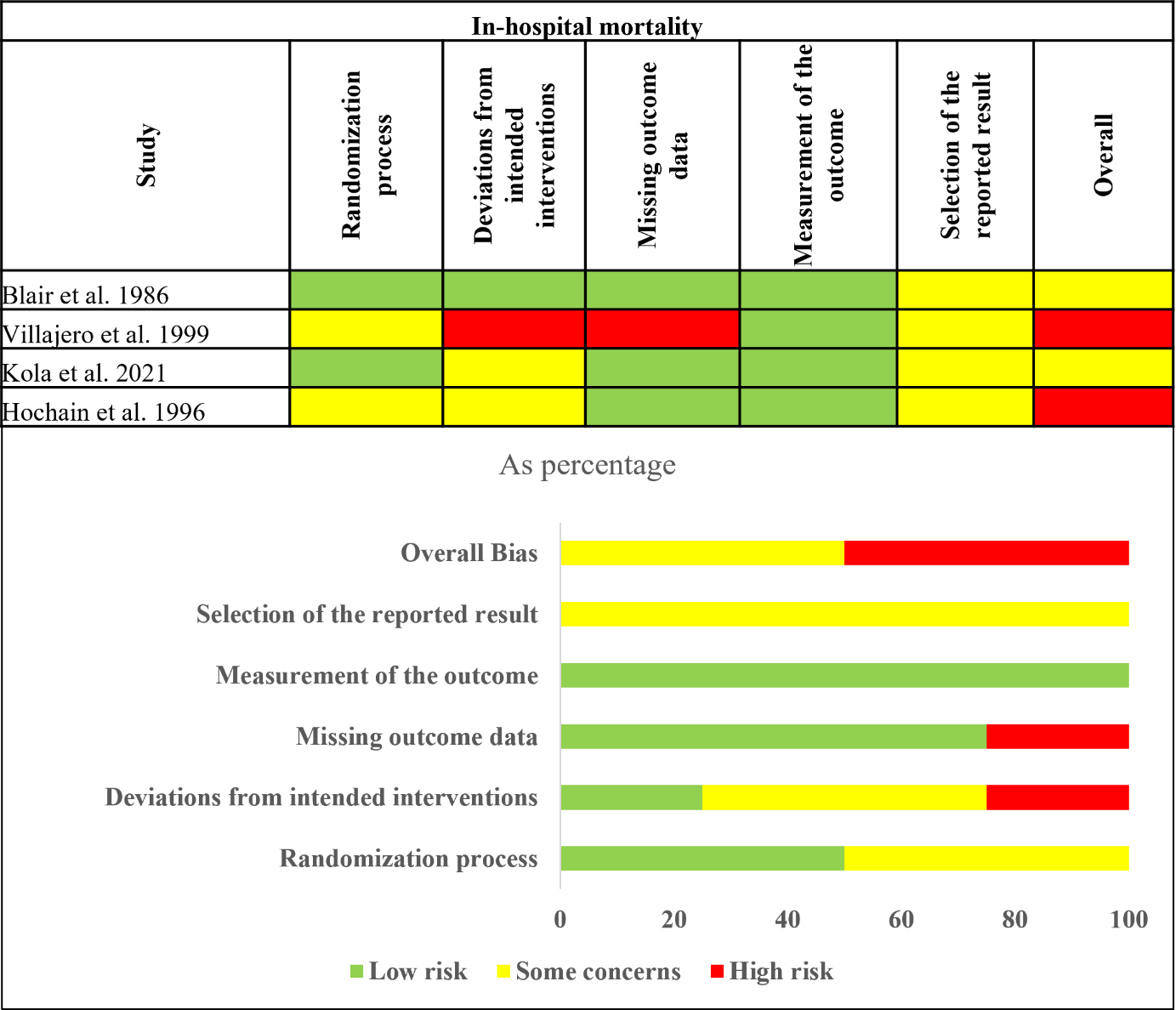


**Figure S17.** Risk of bias assessment at study and at domain level for **in-hospital mortality** (meta-analysis)


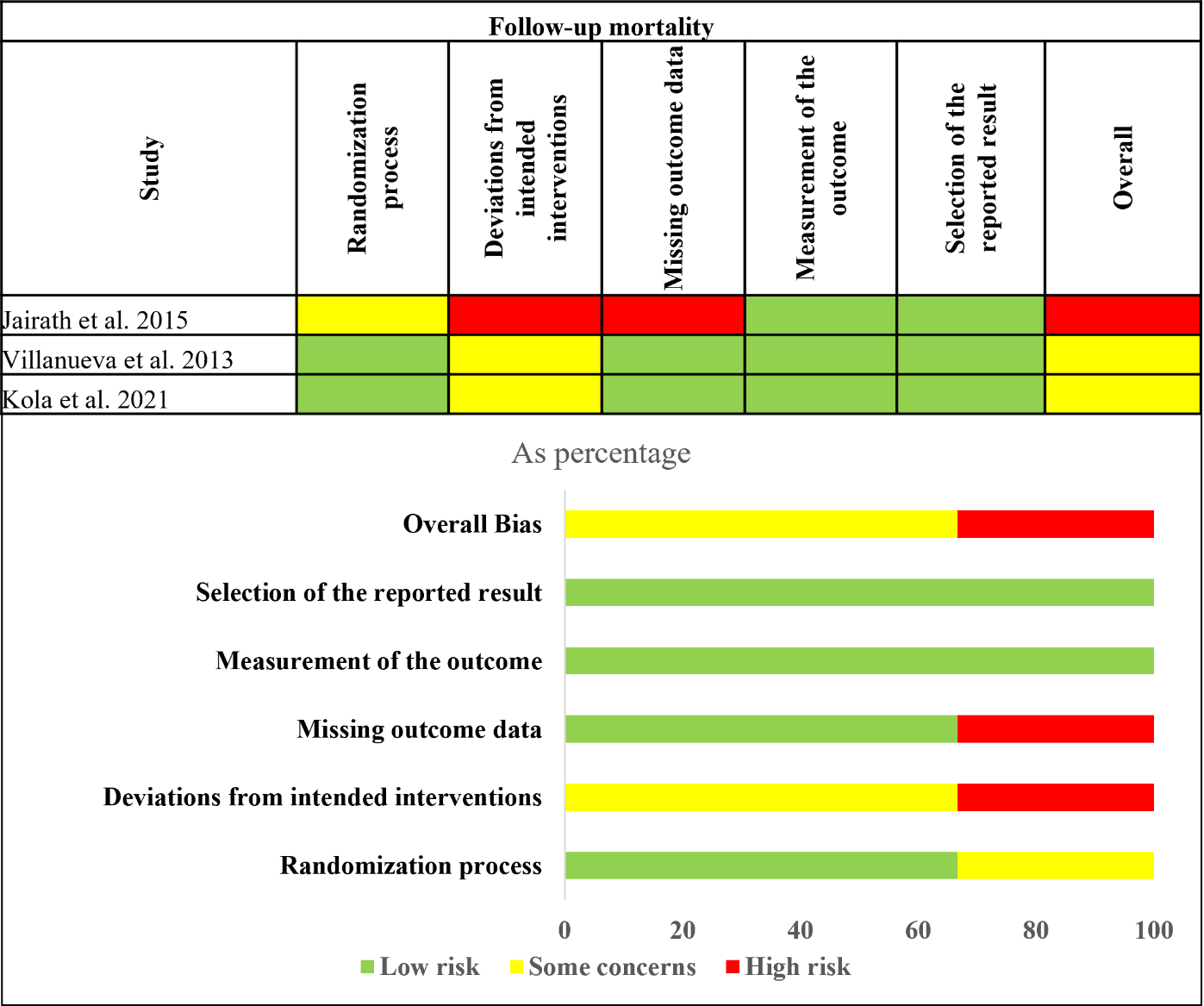


**Figure S18.** Risk of bias assessment at study and at domain level for **follow-up mortality** (meta-analysis)


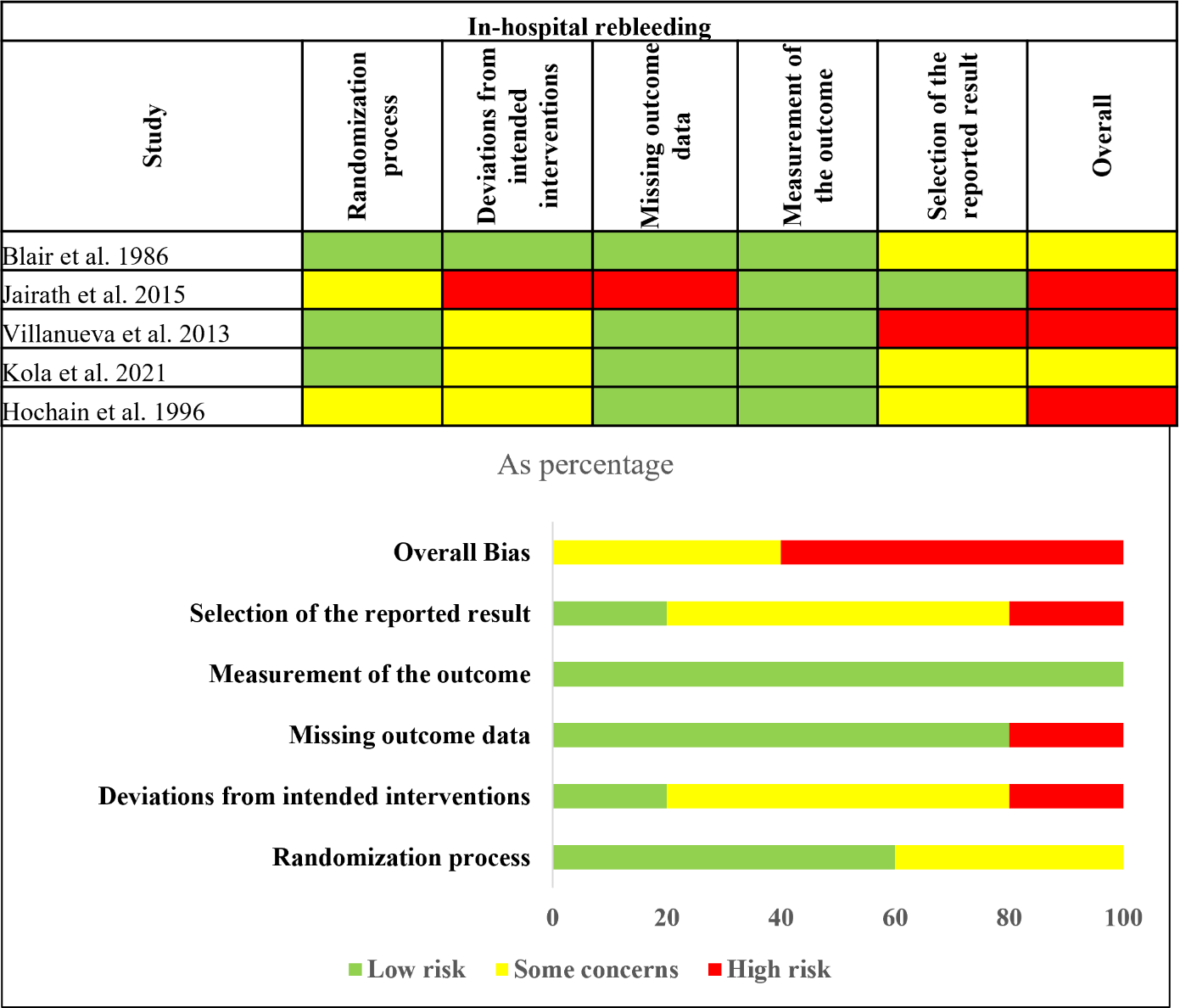


**Figure S19.** Risk of bias assessment at study and at domain level for **in-hospital rebleeding** (meta-analysis)


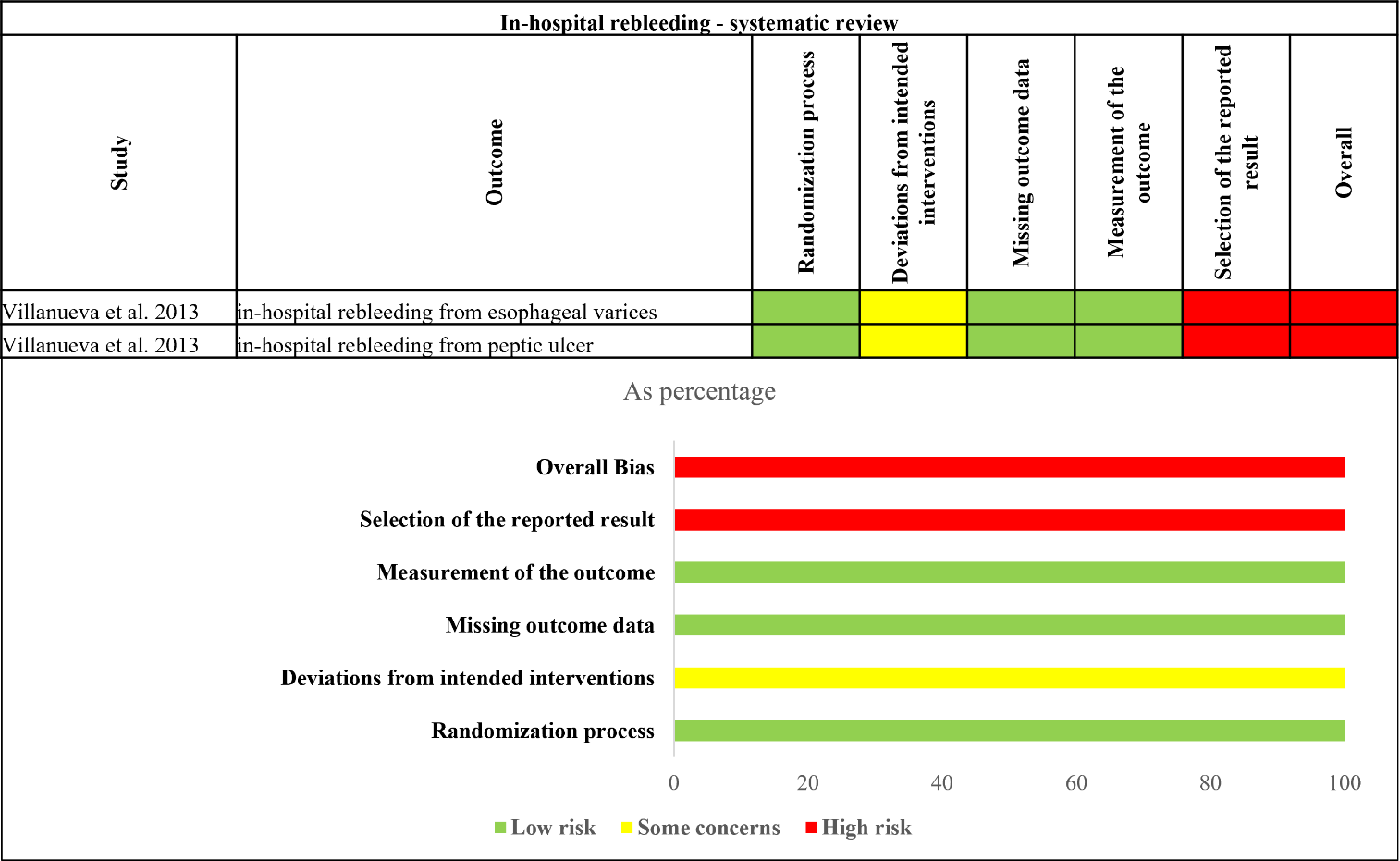


**Figure S20.** Risk of bias assessment at study and at domain level for **in-hospital rebleeding** (systematic review)


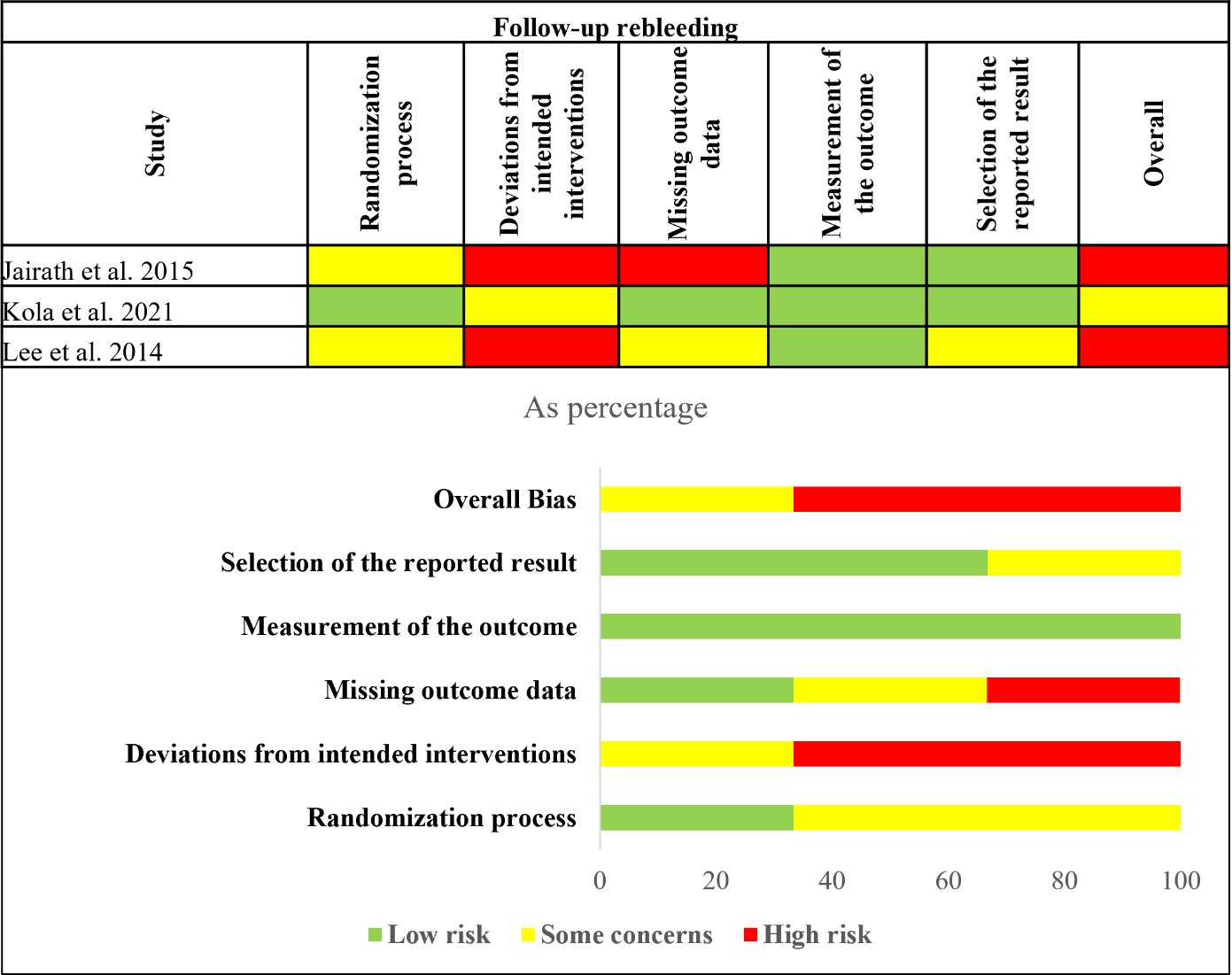


**Figure S21.** Risk of bias assessment at study and at domain level for **follow-up rebleeding** (meta-analysis)


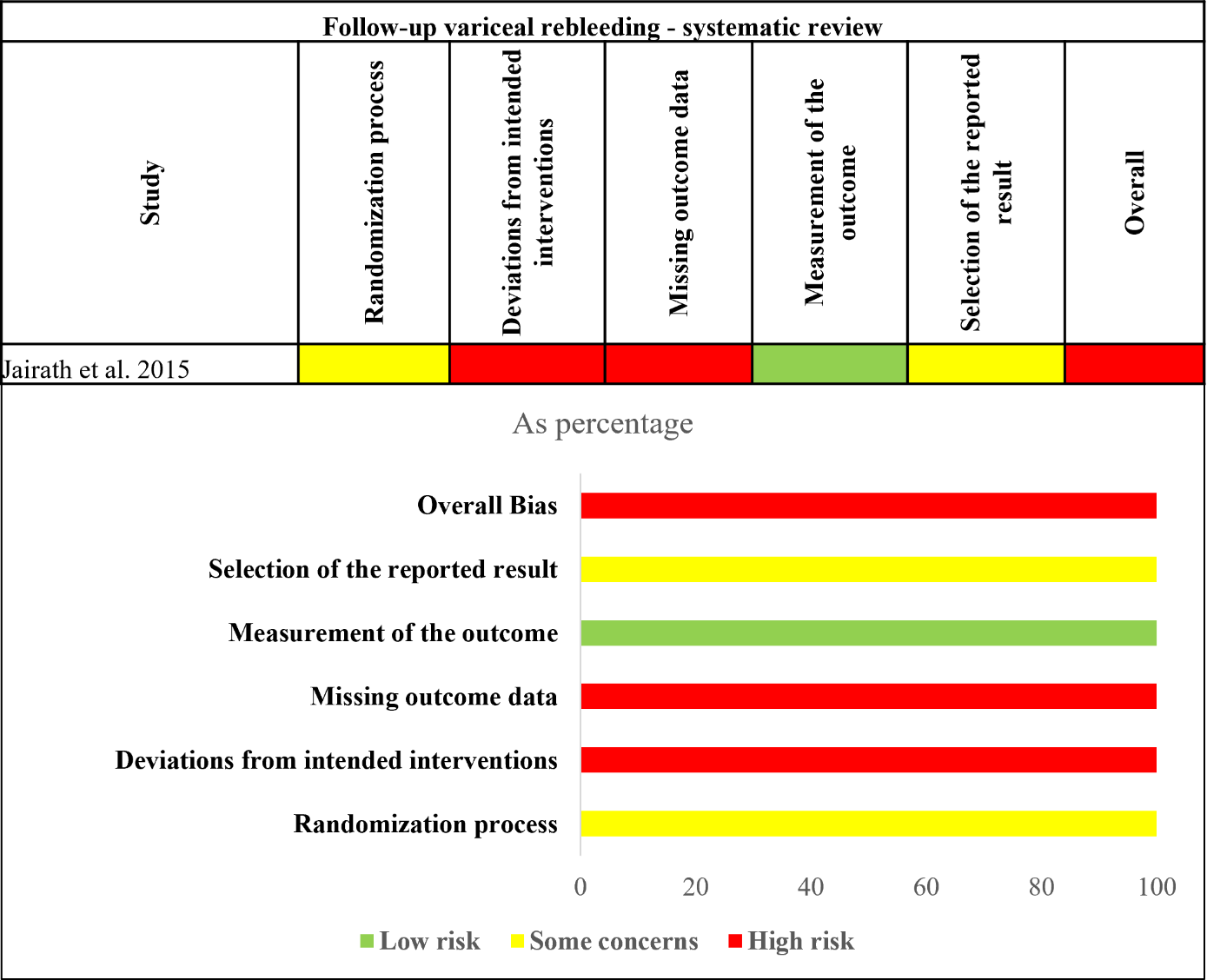


**Figure S22.** Risk of bias assessment at study and at domain level for **follow-up rebleeding** (systematic review)


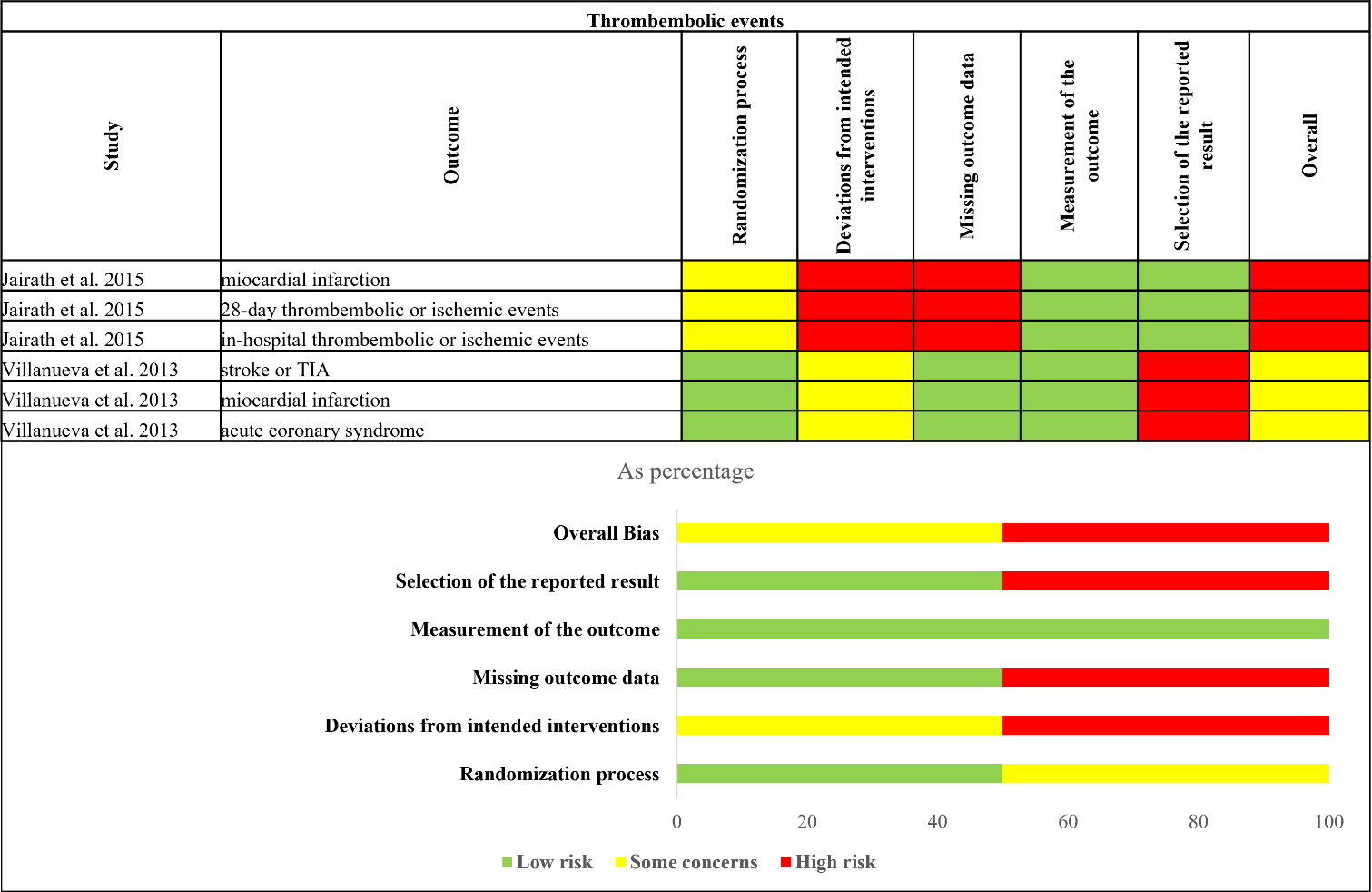


**Figure S23.** Risk of bias assessment at study and at domain level for **thromboembolic events** (systematic review)


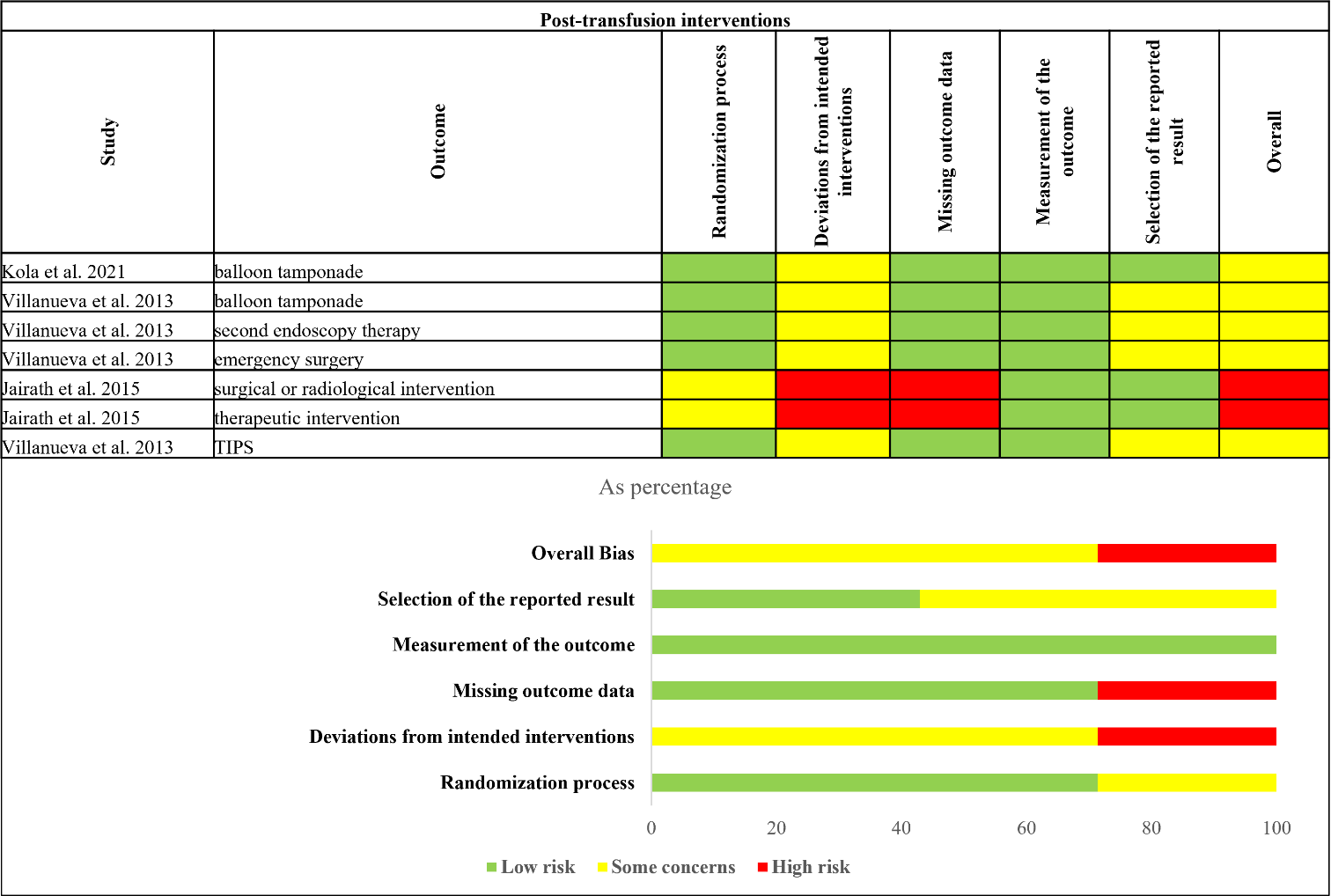


**Figure S24.** Risk of bias assessment at study and at domain level for **post-transfusion interventions** (systematic review)


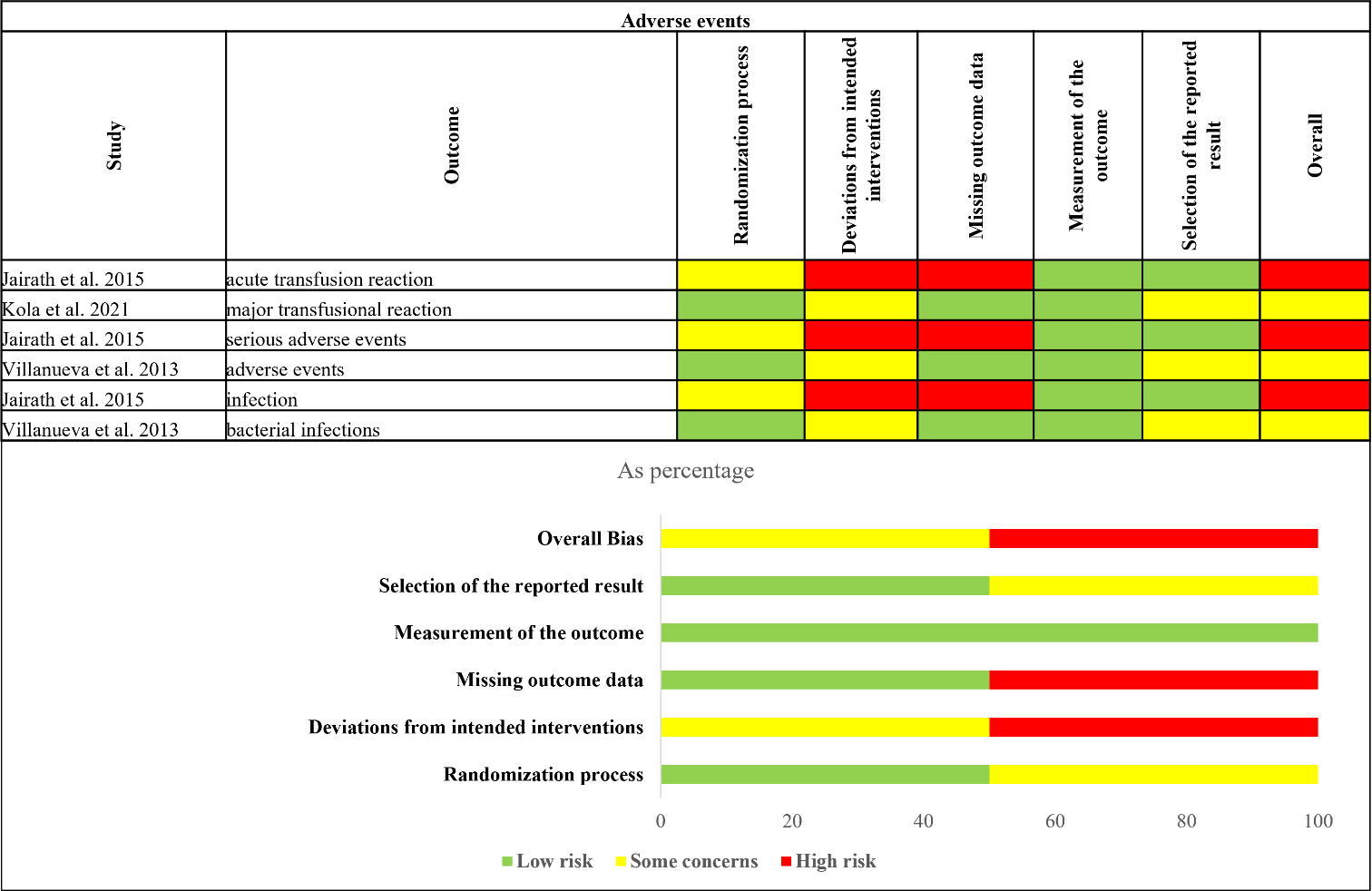


**Figure S25.** Risk of bias assessment at study and at domain level for **adverse events** (systematic review)

**Table S6.** Quality of evidence table for the meta-analysis

| **Restrictive transfusion compared to liberal transfusion in acute upper gastrointestinal bleeding** | | | | | |
| --- | --- | --- | --- | --- | --- |
| **Patient or population:** acute upper gastrointestinal bleeding  **Setting:**  **Intervention:** restrictive transfusion  **Comparison:** liberal transfusion | | | | | |
| **Outcomes** | **№ of participants (studies) Follow-up** | **Certainty of the evidence (GRADE)** | **Relative effect (95% CI)** | **Anticipated absolute effects** | |
|  |  |  |  | **Risk with liberal transfusion** | **Risk difference with restrictive transfusion** |
| Units of red blood cells transfused | 1830 (5 RCTs) | ⨁◯◯◯ Very low^a,b,c,d^ | - | The mean units of red blood cells transfused was **0** units | MD **1.35 units fewer** (2.39 fewer to 0.32 fewer) |
| In-hospital mortality | 391 (4 RCTs) | ⨁◯◯◯ Very low^a,c,d^ | **RR 0.95** (0.46 to 1.94) | 71 per 1,000 | **4 fewer per 1,000** (39 fewer to 67 more) |
| Mortality follow-up: range 28 days to 30 days | 1753 (3 RCTs) | ⨁◯◯◯ Very low^a,d,e^ | **RR 0.71** (0.35 to 1..45) | 83 per 1,000 | **24 fewer per 1,000** (54 fewer to 37 more) |
| In-hospital rebleeding | 1893 (5 RCTs) | ⨁◯◯◯ Very low^a,c,d,f,g^ | **RR 0.67** (0.30 to 1.50) | 135 per 1,000 | **44 fewer per 1,000** (94 fewer to 67 more) |
| Rebleeding follow-up: range 28 days to 45 days | 927 (3 RCTs) | ⨁◯◯◯ Very low^a,d,g^ | **RR 0.75** (0.49 to 1.16) | 97 per 1,000 | **24 fewer per 1,000** (49 fewer to 16 more) |
| Acute kidney injury | 1504 (3 RCTs) | ⨁◯◯◯ Very low^a,c,d,g^ | **RR 0.79** (0.61 to 1.03) | 137 per 1,000 | **29 fewer per 1,000** (53 fewer to 4 more) |
| Length of hospital stay | 1140 (3 RCTs) | ⨁◯◯◯ Very low^a,c,d,g,h^ | - | The mean length of hospital stay was **0** days | MD **0.49 days lower** (1.86 lower to 0.89 higher) |
| ***The risk in the intervention group** (and its 95% confidence interval) is based on the assumed risk in the comparison group and the **relative effect** of the intervention (and its 95% CI).  **CI:** confidence interval; **MD:** mean difference; **RR:** risk ratio | | | | | |
| **GRADE Working Group grades of evidence** **High certainty:** we are very confident that the true effect lies close to that of the estimate of the effect. **Moderate certainty:** we are moderately confident in the effect estimate: the true effect is likely to be close to the estimate of the effect, but there is a possibility that it is substantially different. **Low certainty:** our confidence in the effect estimate is limited: the true effect may be substantially different from the estimate of the effect. **Very low certainty:** we have very little confidence in the effect estimate: the true effect is likely to be substantially different from the estimate of effect. | | | | | |

#### Explanations

a. High proportion of studies carried a moderate or high risk of overall bias.

b. High statistical heterogeneity (I²=95%, CI: 91-97%).

c. Different interventions and controls (different haemoglobin and haematocrit thresholds for transfusion).

d. Publication bias could not be assessed due to the low study number.

e. Overall effect is not precise; the upper part of the CI is close to the line of no effect. Small sample size to show differences between the two groups.

f. Moderate statistical heterogeneity with large confidence interval (I²=42%, CI: 0-79%).

g. Overall effect is not precise; the upper part of the CI crosses the line of no effect. Small sample size to show differences between the two groups.

h. High statistical heterogeneity (I²=79%, CI: 34-94%).

**Table S7.** Quality of evidence table for the systematic review (units of red blood cells transfused, rebleeding)

| **Restrictive transfusion compared to liberal transfusion in acute upper gastrointestinal bleeding** | | | | | |
| --- | --- | --- | --- | --- | --- |
| **Patient or population:** acute upper gastrointestinal bleeding  **Setting:** Transfusion, rebleeding  **Intervention:** restrictive transfusion  **Comparison:** liberal transfusion | | | | | |
| **Outcomes** | **№ of participants (studies) Follow-up** | **Certainty of the evidence (GRADE)** | **Relative effect (95% CI)** | **Anticipated absolute effects** | |
|  |  |  |  | **Risk with liberal transfusion** | **Risk difference with restrictive transfusion** |
| Units of red blood cells transfused | 90 (1 RCT) | ⨁⨁◯◯ Low^a,b^ | - | The mean units of RBC transfused was **0** RBC units | MD **1.8 RBC units lower** (0 to 0 ) |
| Rebleeding from gastrooeasophageal varices | 190 (1 RCT) | ⨁⨁◯◯ Low^a,b^ | **HR 0.50** (0.23 to 0.99) [Rebleeding from gastrooeasophageal varices] | **Low** | |
|  |  |  |  | 0 per 1,000 | **-- per 1,000** (-- to --) |
| Rebleeding from gastrooeasophageal varices follow-up: 28 days | 43 (1 RCT) | ⨁⨁◯◯ Low^a,b^ | not estimable | **Low** | |
|  |  |  |  | 0 per 1,000 | **0 fewer per 1,000** (0 fewer to 0 fewer) |
| Rebleeding from peptic ulcer | 437 (1 RCT) | ⨁⨁◯◯ Low^a,b^ | **HR 0.63** (0.37 to 1.07) [Rebleeding from peptic ulcer] | **Low** | |
|  |  |  |  | 0 per 1,000 | **-- per 1,000** (-- to --) |
| ***The risk in the intervention group** (and its 95% confidence interval) is based on the assumed risk in the comparison group and the **relative effect** of the intervention (and its 95% CI).  **CI:** confidence interval; **HR:** hazard Ratio; **MD:** mean difference | | | | | |
| **GRADE Working Group grades of evidence** **High certainty:** we are very confident that the true effect lies close to that of the estimate of the effect. **Moderate certainty:** we are moderately confident in the effect estimate: the true effect is likely to be close to the estimate of the effect, but there is a possibility that it is substantially different. **Low certainty:** our confidence in the effect estimate is limited: the true effect may be substantially different from the estimate of the effect. **Very low certainty:** we have very little confidence in the effect estimate: the true effect is likely to be substantially different from the estimate of effect. | | | | | |

#### Explanations

a. The study carried a high risk of overall bias.

b. Publication bias could not be assessed due to the low study number.

**Table S8.** Quality of evidence table for the systematic review (thromboembolic events)

| **Restrictive transfusion compared to liberal transfusion in acute upper gastrointestinal bleeding** | | | | | |
| --- | --- | --- | --- | --- | --- |
| **Patient or population:** acute upper gastrointestinal bleeding  **Setting:** Thrombembolic events  **Intervention:** restrictive transfusion  **Comparison:** liberal transfusion | | | | | |
| **Outcomes** | **№ of participants (studies) Follow-up** | **Certainty of the evidence (GRADE)** | **Relative effect (95% CI)** | **Anticipated absolute effects** | |
|  |  |  |  | **Risk with liberal transfusion** | **Risk difference with restrictive transfusion** |
| Thrombembolic events | 640 (1 RCT) | ⨁⨁◯◯ Low^a,b^ | not estimable | 55 per 1,000 | **55 fewer per 1,000** (55 fewer to 55 fewer) |
| Thrombembolic event follow-up: 28 days | 640 (1 RCT) | ⨁⨁◯◯ Low^a,b^ | not estimable | 60 per 1,000 | **60 fewer per 1,000** (60 fewer to 60 fewer) |
| Myocardial infarction | 1471 (2 RCTs) | ⨁◯◯◯ Very low^b,c,d^ | not estimable | 13 per 1,000 | **13 fewer per 1,000** (13 fewer to 13 fewer) |
| Acute coronary syndrome | 889 (1 RCT) | ⨁⨁⨁◯ Moderate^b,e^ | not estimable | 29 per 1,000 | **29 fewer per 1,000** (29 fewer to 29 fewer) |
| Stroke or transient ischemic attack | 889 (1 RCT) | ⨁⨁⨁◯ Moderate^b,e^ | not estimable | 13 per 1,000 | **13 fewer per 1,000** (13 fewer to 13 fewer) |
| ***The risk in the intervention group** (and its 95% confidence interval) is based on the assumed risk in the comparison group and the **relative effect** of the intervention (and its 95% CI).  **CI:** confidence interval | | | | | |
| **GRADE Working Group grades of evidence** **High certainty:** we are very confident that the true effect lies close to that of the estimate of the effect. **Moderate certainty:** we are moderately confident in the effect estimate: the true effect is likely to be close to the estimate of the effect, but there is a possibility that it is substantially different. **Low certainty:** our confidence in the effect estimate is limited: the true effect may be substantially different from the estimate of the effect. **Very low certainty:** we have very little confidence in the effect estimate: the true effect is likely to be substantially different from the estimate of effect. | | | | | |

#### Explanations

a. The study carried a high risk of overall bias.

b. Publication bias could not be assessed due to the low study number.

c. The studies carried a moderate and high risk of overall bias.

d. Different interventions and controls (different haemoglobin thresholds for transfusion).

e. The study carried a moderate risk of overall bias.

**Table S9.** Quality of evidence table for the systematic review (post-transfusion interventions)

| **Restrictive transfusion compared to liberal transfusion in acute upper gastrointestinal bleeding** | | | | | |
| --- | --- | --- | --- | --- | --- |
| **Patient or population:** acute upper gastrointestinal bleeding  **Setting:** post-transfusion interventions  **Intervention:** restrictive transfusion  **Comparison:** liberal transfusion | | | | | |
| **Outcomes** | **№ of participants (studies) Follow-up** | **Certainty of the evidence (GRADE)** | **Relative effect (95% CI)** | **Anticipated absolute effects** | |
|  |  |  |  | **Risk with liberal transfusion** | **Risk difference with restrictive transfusion** |
| Therapeutic intervention | 640 (1 RCT) | ⨁⨁◯◯ Low^a,b^ | not estimable | 376 per 1,000 | **376 fewer per 1,000** (376 fewer to 376 fewer) |
| Emergency surgery | 437 (1 RCT) | ⨁⨁⨁◯ Moderate^b,c^ | not estimable | 57 per 1,000 | **57 fewer per 1,000** (57 fewer to 57 fewer) |
| Surgical or radiological intervention | 640 (1 RCT) | ⨁⨁◯◯ Low^a,b^ | not estimable | 29 per 1,000 | **29 fewer per 1,000** (29 fewer to 29 fewer) |
| TIPS | 277 (1 RCT) | ⨁⨁⨁◯ Moderate^b,c^ | not estimable | 109 per 1,000 | **109 fewer per 1,000** (109 fewer to 109 fewer) |
| Balloon tamponade | 501 (2 RCTs) | ⨁⨁⨁◯ Moderate^b,d^ | not pooled | not pooled | not pooled |
| Second endoscopy | 437 (1 RCT) | ⨁⨁⨁◯ Moderate^b,c^ | not estimable | 124 per 1,000 | **124 fewer per 1,000** (124 fewer to 124 fewer) |
| ***The risk in the intervention group** (and its 95% confidence interval) is based on the assumed risk in the comparison group and the **relative effect** of the intervention (and its 95% CI).  **CI:** confidence interval | | | | | |
| **GRADE Working Group grades of evidence** **High certainty:** we are very confident that the true effect lies close to that of the estimate of the effect. **Moderate certainty:** we are moderately confident in the effect estimate: the true effect is likely to be close to the estimate of the effect, but there is a possibility that it is substantially different. **Low certainty:** our confidence in the effect estimate is limited: the true effect may be substantially different from the estimate of the effect. **Very low certainty:** we have very little confidence in the effect estimate: the true effect is likely to be substantially different from the estimate of effect. | | | | | |

#### Explanations

a. The study carried a high risk of overall bias.

b. Publication bias could not be assessed due to the low study number.

c. The study carried a moderate risk of overall bias.

d. The studies carried a moderate risk of overall bias.

**Table S10.** Quality of evidence table for the systematic review (adverse events)

| **Restrictive transfusion compared to liberal transfusion in acute upper gastrointestinal bleeding** | | | | | |
| --- | --- | --- | --- | --- | --- |
| **Patient or population:** acute upper gastrointestinal bleeding  **Setting:** Adverse events  **Intervention:** restrictive transfusion  **Comparison:** liberal transfusion | | | | | |
| **Outcomes** | **№ of participants (studies) Follow-up** | **Certainty of the evidence (GRADE)** | **Relative effect (95% CI)** | **Anticipated absolute effects** | |
|  |  |  |  | **Risk with liberal transfusion** | **Risk difference with restrictive transfusion** |
| Transfusional reaction | 864 (2 RCTs) | ⨁⨁◯◯ Low^a,b^ | not pooled | not pooled | not pooled |
| Serious adverse event | 640 (1 RCT) | ⨁⨁◯◯ Low^b,c^ | not estimable | 217 per 1,000 | **217 fewer per 1,000** (217 fewer to 217 fewer) |
| Adverse event | 889 (1 RCT) | ⨁⨁⨁◯ Moderate^b,d^ | not estimable | 481 per 1,000 | **481 fewer per 1,000** (481 fewer to 481 fewer) |
| Infection | 1529 (2 RCTs) | ⨁⨁⨁◯ Moderate^a,b^ | not pooled | not pooled | not pooled |
| ***The risk in the intervention group** (and its 95% confidence interval) is based on the assumed risk in the comparison group and the **relative effect** of the intervention (and its 95% CI).  **CI:** confidence interval | | | | | |
| **GRADE Working Group grades of evidence** **High certainty:** we are very confident that the true effect lies close to that of the estimate of the effect. **Moderate certainty:** we are moderately confident in the effect estimate: the true effect is likely to be close to the estimate of the effect, but there is a possibility that it is substantially different. **Low certainty:** our confidence in the effect estimate is limited: the true effect may be substantially different from the estimate of the effect. **Very low certainty:** we have very little confidence in the effect estimate: the true effect is likely to be substantially different from the estimate of effect. | | | | | |

#### Explanations

a. The studies carried a moderate and high risk of overall bias.

b. Publication bias could not be assessed due to the low study number.

c. The study carried a high risk of overall bias.

d. The study carried a moderate risk of overall bias.
